# Explosive radiation and spatial expansion across the cold environments of the Old World in an avian family

**DOI:** 10.1002/ece3.3136

**Published:** 2017-07-06

**Authors:** Baoyan Liu, Per Alström, Urban Olsson, Jon Fjeldså, Qing Quan, Kees C. S. Roselaar, Takema Saitoh, Cheng‐te Yao, Yan Hao, Wenjuan Wang, Yanhua Qu, Fumin Lei

**Affiliations:** ^1^ Key Laboratory of Zoological Systematics and Evolution Institute of Zoology Chinese Academy of Sciences Beijing China; ^2^ University of Chinese Academy of Sciences Beijing China; ^3^ Department of Animal Ecology Evolutionary Biology Centre Uppsala University Uppsala Sweden; ^4^ Swedish Species Information Centre Swedish University of Agricultural Sciences Uppsala Sweden; ^5^ Systematics and Biodiversity Department of Biology and Environmental Sciences University of Gothenburg Göteborg Sweden; ^6^ Centre for Macroecology, Evolution and Climate Zoological Museum University of Copenhagen Copenhagen Denmark; ^7^ Naturalis Biodiversity Center Leiden The Netherlands; ^8^ Yamashina Institute for Ornithology Abiko Chiba Japan; ^9^ High‐Altitude Experimental Station Endemic Species Research Institute COA Chi‐chi Taiwan , China; ^10^ Center for Watershed Ecology Institute of Life Science and Ministry of Education Key Laboratory of Poyang Lake Environment and Resource Utilization Nanchang University Nanchang China

**Keywords:** hard polytomy, *Prunella*, secondary contact, speciation, sympatry

## Abstract

Our objective was to elucidate the biogeography and speciation patterns in an entire avian family, which shows a complex pattern of overlapping and nonoverlapping geographical distributions, and much variation in plumage, but less in size and structure. We estimated the phylogeny and divergence times for all of the world's species of *Prunella* based on multiple genetic loci, and analyzed morphometric divergence and biogeographical history. The common ancestor of *Prunella* was present in the Sino‐Himalayan Mountains or these mountains and Central Asia–Mongolia more than 9 million years ago (mya), but a burst of speciations took place during the mid‐Pliocene to early Pleistocene. The relationships among the six primary lineages resulting from that differentiation are unresolved, probably because of the rapid radiation. A general increase in sympatry with increasing time since divergence is evident. With one exception, species in clades younger than c. 3.7 my are allopatric. Species that are widely sympatric, including the most recently diverged (2.4 mya) sympatric sisters, are generally more divergent in size/structure than allo‐/parapatric close relatives. The distributional pattern and inferred ages suggest divergence in allopatry and substantial waiting time until secondary contact, likely due to competitive exclusion. All sympatrically breeding species are ecologically segregated, as suggested by differences in size/structure and habitat. Colonizations of new areas were facilitated during glacial periods, followed by fragmentation during interglacials—contrary to the usual view that glacial periods resulted mainly in fragmentations.

## 1 INTRODUCTION

Speciation concerns the splitting of one lineage into two (or more), with gradual divergence in various traits, eventually leading to reproductive isolation between these sister lineages (Coyne & Orr, 2004; Price, 2008). Price (2008) reviewed speciation in birds and suggested the following general sequence of events: (1) range expansion; (2) restrictions to gene flow resulting from range expansion; (3) divergence in various traits that contribute to reproductive and ecological isolation; (4) establishment of sympatry (secondary contact), possibly aided by reinforcement of premating isolation due to low fitness of hybrids and/or ecological character displacement resulting from competition. However, the magnitude of divergence and the relative importance of different traits (structure, plumage, vocalizations, and behavior) as well as time required for reproductive isolation is poorly known. Grant and Grant (2008) stressed the importance of ecological divergence under natural selection during the initial allopatric phase, and noted that barriers to interbreeding could arise as byproducts of adaptive divergence. For example, divergence in overall size and, especially, bill size resulting from adaptation to different food resources has been shown to affect mate choice directly or indirectly by causing vocal divergence (Grant, Grant, & Petren, 2000; Ratcliffe & Grant, 1983). Competitive exclusion has been suggested to be the main factor limiting the build‐up of sympatric communities (e.g., Pigot & Tobias, 2013; Price et al., 2014), with substantial divergence in allopatry being required prior to establishment of sympatry (e.g., Pigot & Tobias, 2013; Price & Kirkpatrick, 2009; Price et al., 2014; Webb, Ackerly, McPeek, & Donoghue, 2002).

Most studies of speciation and biogeography in temperate‐zone birds have focused on describing the historical population structure of single species, or of a few closely related species (e.g., Drovetski, Raković, Semenov, Fadeev, & Red'kin, 2014; Drovetski et al., 2004; Haring, Gamauf, & Kryukov, 2007; Irwin, Bensch, & Price, 2001; Li et al., 2016; Milá, McCormack, Castaneda, Wayne, & Smith, 2007; Ödeen & Björklund, 2003; Pavlova, Rohwer, Drovetski, & Zink, 2006; Pavlova, Zink, & Rohwer, 2005; Pavlova, Zink, Rohwer, Koblik, et al., 2005; Qvarnström, Rice, & Ellegren, 2010; Saitoh et al., 2010; Song et al., 2016; Zhao et al., 2012; Zink, Pavlova, Drovetski, & Rohwer, 2008; Zink et al., 2003). In order to find general patterns, there is a need to build complete species level phylogenies for larger taxonomic groups, with species in different stages of the speciation process.

**Figure 1:**
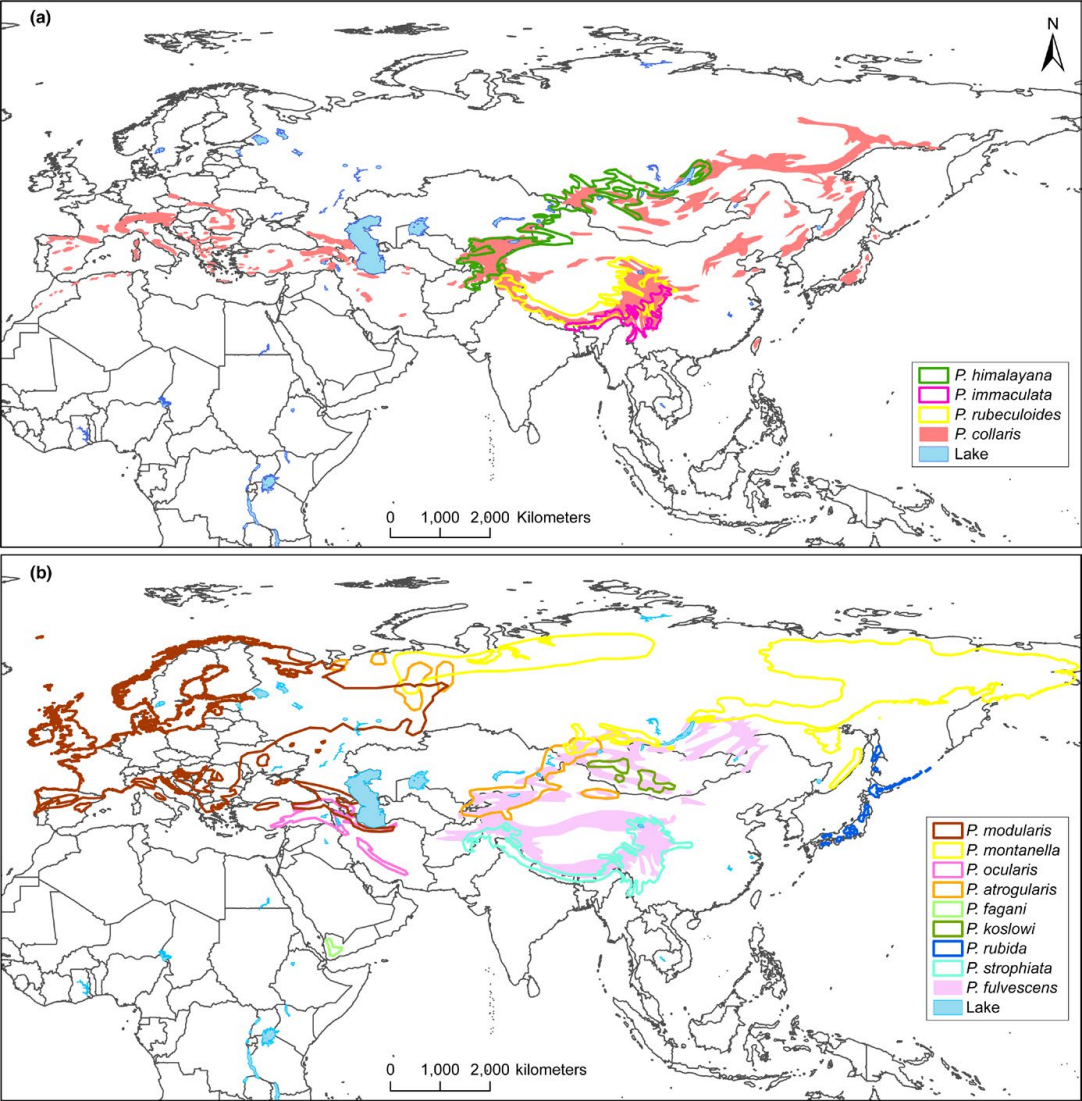
Distributions based on compilation by C.S.R. (unpublished), Gombobaatar et al. (2011), and Y. Red'kin, E. Koblik & A. Mosalov (in prep.): (a) the four species representing the earliest branches in the phylogeny; (b) the species in clade C (Figures 2 and 3). Note extensive sympatry of five species in the eastern Himalayas to central China and four species in Central Asia, and marginal sympatry of three species in the Ural Mountains

**Figure 2:**
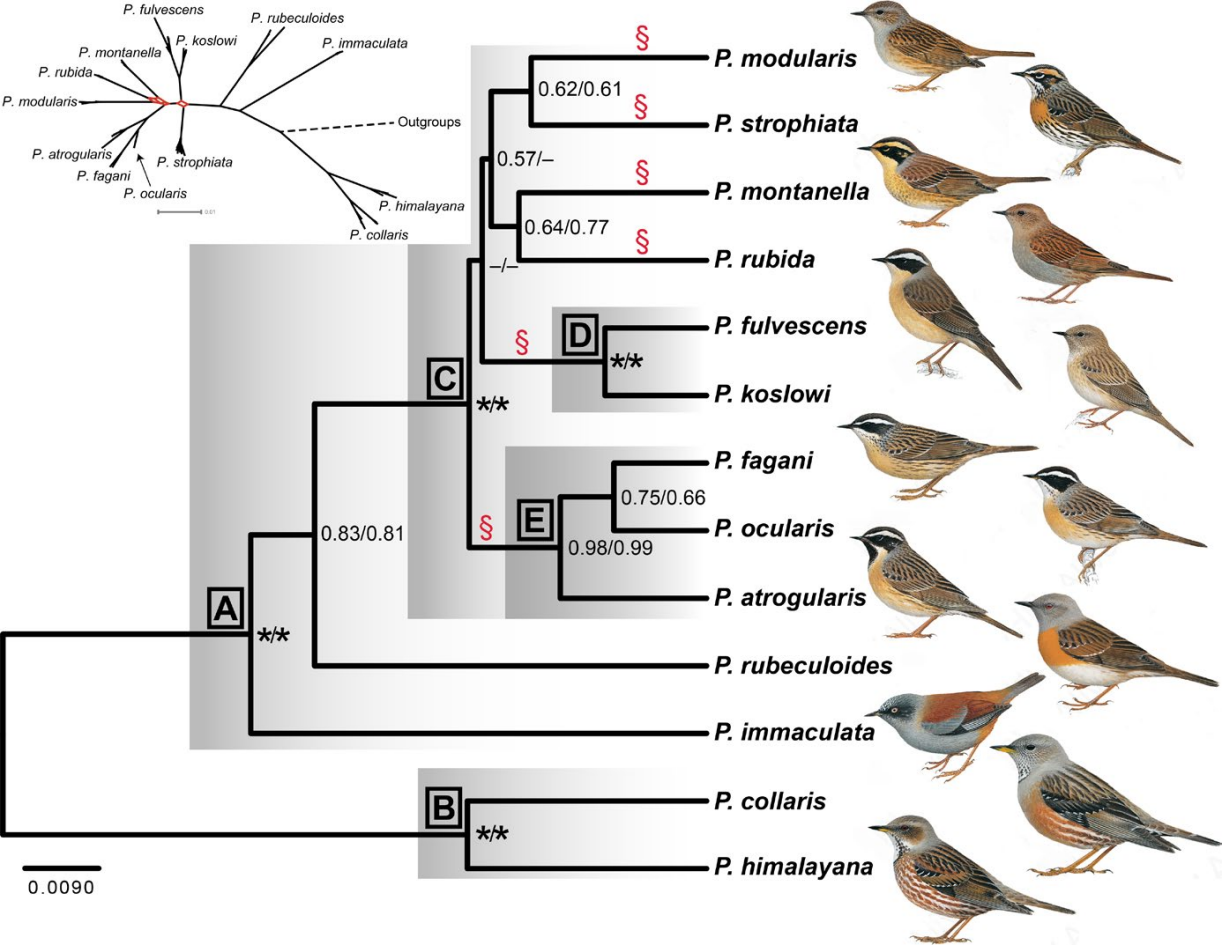
Phylogeny based on 10 nuclear and two mitochondrial loci, inferred by *BEAST. Posterior probabilities (PP) indicated at nodes (11/18 partitions); asterisk PP 1.00. Letters a–e refer to clades discussed in the text. § indicates primary lineages in clade C resulting from explosive radiation. Insert: Network based on Bayesian Inference analysis of concatenated sequences analyzed in 18 partitions (threshold 0.10); branches with network‐like pattern indicated in red. Illustrations by Ren Hathway, from del Hoyo, Elliott, & Christie (2005), reproduced by permission from Lynx Edicions

We here focus on the avian family Prunellidae (accentors). This is a close‐knit group, with all 13 currently recognized species placed in the genus *Prunella* (Gill & Donsker, 2016). The entire radiation falls within the Palearctic region (Figure 1). Up to five species breed sympatrically in the eastern Sino‐Himalayan Mountains. Several others are allo‐/parapatrically distributed, with a number of latitudinal and longitudinal range disjunctions. Some of these disjunctions can be related to the patchiness of the preferred habitat, as most accentors breed in alpine environments and near the upper timberline in high mountains. Accentors are mostly resident, descending to lower altitudes in winter, but northerly breeding populations are wholly or mainly migratory (Hatchwell, 2005; Snow & Perrins, 1998). All accentors are similar in structure, including bill size and shape, although there are differences in size (Figure 2). There is considerably more variation in plumage, especially head pattern (Figure 2), although sexes are basically similar in plumage (Hatchwell, 2005; Snow & Perrins, 1998).

An analysis of the phylogeny of all *Prunella* species based on the mitochondrial ND2 and Z‐linked ACO1I9 was recently published, with the aim to test the role of vicariance in montane regions (Drovetski et al., 2013). This suggested a deep split between *P. collaris* + *P. himalayana* and the others, dated to 7.31 million years ago (mya), successive splits of *P. immaculata* and *P. rubeculoides*, and the rest of the species partitioned into an eastern and a western clade rapidly diversifying from c. 2.1 mya.

In this study, we analyze a considerably larger number of loci than Drovetski et al. (2013), in order to obtain a more well‐founded phylogeny, and use a somewhat different geographical sampling, with more samples from the Eastern Palearctic and fewer from the Western Palearctic. We also analyze data on morphology, ecology, and geographical distributions, to try to answer questions about where and when the species in this family evolved, and which factors have shaped their current distributions and the evolution of sympatric breeding ranges.

## 2 METHODS

### 2.1 Sample collection

We obtained tissue, blood, feathers, or toepads from museum specimens from all of the species in the genus *Prunella*. Some sequences were also downloaded from GenBank. See Table S1.

### 2.2 DNA extraction and sequencing

For the *Prunella* and four outgroup species, we sequenced two mitochondrial genes, three Z‐linked loci and seven autosomal loci. Information about sequences and primers is given in Table S2. All new sequences have been deposited in GenBank (Table S1).

### 2.3 Phylogeny

Trees were estimated by Bayesian inference (BI) using MrBayes 3.2 (Huelsenbeck & Ronquist, 2001; Ronquist & Huelsenbeck, 2003) using different data partitioning schemes: (1) all loci were analyzed separately (single‐locus analyzes, SLAs); (2) sequences were concatenated and partitioned by (a) locus (in total 12 partitions) or (b) locus and, for the coding sequences, codon (in total 18 partitions), or (c) unpartitioned. In order to estimate divergence times, the mitochondrial cytochrome *b* (cyt*b*) data were analyzed in BEAST version 1.8.2 (Drummond, Suchard, Xie, & Rambaut, 2012), with an uncorrelated lognormal relaxed clock (Drummond, Ho, Phillips, & Rambaut, 2006) with a mean clock rate of 2.1%/my (Weir & Schluter, 2008). As analyzes of cyt*b* on its own inferred *P. immaculata* and *P. rubeculoides* to be sisters, with strong support, in conflict with the results from the 18‐partition multilocus and *BEAST analyzes (see below and Section 2.1), we also ran analyzes with the topology constrained to match the well‐supported clades in the 18‐partition and *BEAST trees. Integrative species tree estimation was performed using *BEAST (Heled & Drummond, 2010). The concatenated data were also analyzed by maximum‐likelihood bootstrapping (MLBS; 1,000 replicates) in RAxML‐HPC2 version 8.0.0 (Stamatakis, 2006) as well as parsimony bootstrapping (MPBS; 1,000 replicates) in PAUP* (Swofford, 2002). Trees resulting from concatenation analyzes were also explored in SplitsTree4 (Huson & Bryant, 2006, 2016). See Appendix S1 for details of phylogenetic analyzes.

### 2.4 Biogeography

To reconstruct the historical biogeography of *Prunella*, we coded six geographical regions based on the accentors’ distributions: “Western Palearctic” (A), Western Asia (B), Central Asia and Mongolia (C), “Eastern Palearctic” (mainly Siberia and Russian Far East; D), Japan (E), and Sino‐Himalayan Mountains (Himalayas + Central China; F). We then used the DEC model implemented in the software Reconstruct Ancestral State in Phylogenies 3.1 (RASP; Yu, Harris, Blair, & He, 2015). Dispersal constraints were applied based on assumed probabilities of dispersal between different areas (based on geographical proximity) (Appendix Table S3). To account for topological uncertainty (“S‐DEC’), the results were summed over 80,000 trees obtained from the posterior distribution of a BEAST analysis of all loci concatenated for one individual per species (see above; 20,000 trees discarded as “burn‐in”).

### 2.5 Morphometrics

Measurements of wing length (flattened and stretched), tail length (with dividers inserted to base of central rectrices), and bill length (to skull) were taken in museum collections (Table S4). Nearly, all of the measurements were taken by the same person (C.S.R.), with some complementary measurements taken by P.A. and Peng He. A principal component analysis (PCA) was carried out in SPSS v. 22.0 (IBM Corp.).

## 3 RESULTS

### 3.1 Phylogeny

#### 3.1.1 Sequence characteristics

Sequence data were obtained from three to six individuals per species, except for *P. ocularis*, for which only one sample was available, and for *P. fagani*, for which only two samples were available. It seems possible that the divergence between the two *P. fagani* sequences has been exaggerated due to difficulties of obtaining good sequences from these old museum specimens. See Appendix S1 and Table S1 for further details.

#### 3.1.2 Single‐locus analyzes

The trees based on SLAs varied in resolution and support. In the mitochondrial trees, all of the species were monophyletic, but most relationships among species were uncertain. The trees based on nuclear loci were less resolved and supported, and the sequences did not sort entirely according to species in any of them. See Appendix S1 and Figure. S1 for further details.

#### 3.1.3 Concatenation analyzes

The tree based on the complete concatenated dataset analyzed in 18 partitions (12 loci, three further partitioned by codon, mixed + Γ + I; hereafter concat 18p; Figure S3) had significantly higher likelihood than the trees based on the other partition schemes (Table S5). It was fully resolved with respect to interspecific relationships except for one trichotomy within clade C. Clades A–C were strongly supported. However, within clade C, only clades D and E were strongly supported, whereas other interspecific relationships had low BI and MLBS support. In contrast, the tree based on the complete concatenated dataset analyzed in 12 locus‐specific partitions (hereafter concat12p; Figure S4) was fully resolved, and all except one of the interspecific nodes had PP ≥.95 (one PP .91). However, three of the nodes with PP .91, .95 and .99, respectively, had MLBS and MPBS <50%. Other analyzes of this dataset differed only slightly from the concat18p tree (see Appendix S1 and Figures [Link], [Link], [Link] for further details). A consensus tree with threshold set to 0.10 in SplitsTree4 showed much network‐like pattern at the base of clade C (Figure 2).

#### 3.1.4 *BEAST tree

The tree inferred by *BEAST (Figure 2) strongly supported clades A–E, whereas the other relationships within clade C were poorly supported. It agreed perfectly with the concat18p tree, if all poorly supported clades were collapsed in both these trees.

### 3.2 Molecular clock dating

**Figure 3:**
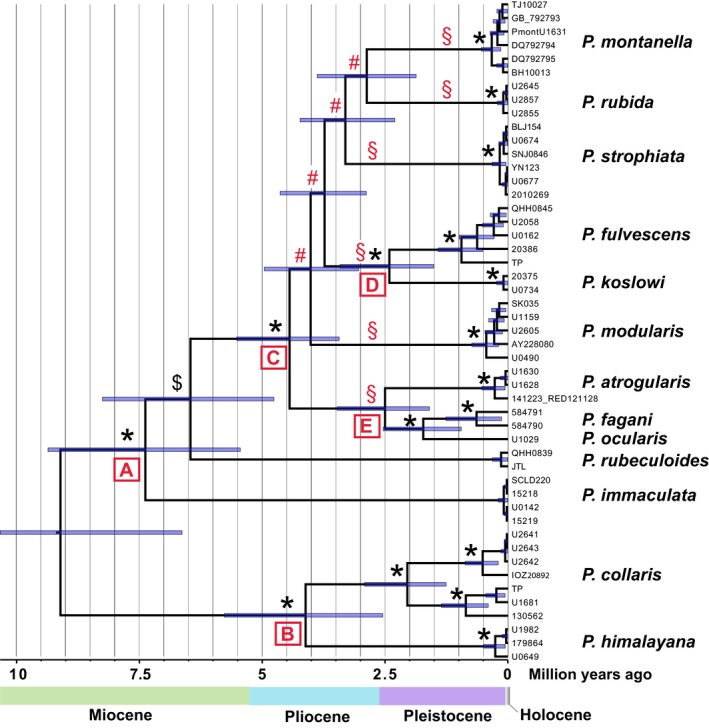
Chronogram based on cytochrome *b* sequences and a relaxed molecular clock (2.1%/MY), inferred by Bayesian inference (BEAST); topology constrained to agree with that in Figure 2 with respect to position of *Prunella rubeculoides* (node indicated by $). Horizontal bars at nodes are 95% highest posterior density intervals for node ages. Posterior probabilities (PP) 1.00 are indicated above the nodes by an asterisk; # indicates PP .28–.87. § indicates primary lineages in clade C

The *Prunella* cyt*b* chronogram (Figure 3) suggested that the deepest split, between clades A and B, was c. 9 million years (my) old (95% HPD 6.6–11.8 million years ago [mya]), and that the species within clade C diverged within the time period c. 4.4–1.7 mya (combined 95% HPD 0.95–5.5 mya), that is mainly during the mid‐Pliocene to early Pleistocene. Deep intraspecies divergences were found in especially *P. collaris*, but also in *P. fulvescens* and, as noted above to be likely spurious, in *P. fagani*.

### 3.3 Biogeography and speciation

The results from the RASP analysis are shown in Figure S7. The ancestor of *Prunella* was inferred to have been present in the Sino‐Himalayan Mountains or the Sino‐Himalayan Mountains and Central Asia–Mongolia, with later expansion out of this region.

**Figure 4:**
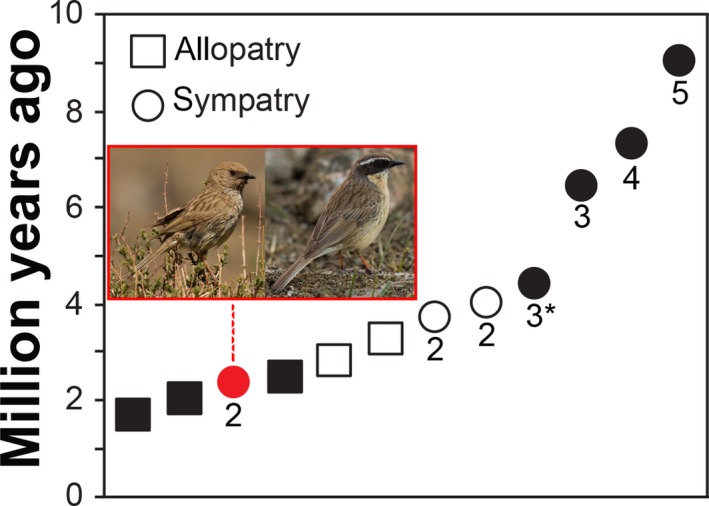
Geographical overlap versus divergence times for all clades with two or more species (plus the deep divergence within *Prunella collaris*; cf. Figure 3), demonstrating long waiting time to sympatry, except in one species pair, where the plumage and structural divergence is unusually high for recently diverged species (cf. also Figures 2 and 5). The symbols are spread out along the *X*‐axis for clarity. Numbers next to symbols refer to maximum number of sympatric species in any one area; 3* indicates three mostly parapatric species, with very marginal geographical overlap; the red circle indicates the sympatry between the sister species *P. koslowi* and *P. fulvescens*. Unfilled symbols indicate clades with poor support (marked by # in Figure 3). Photos of *P. koslowi* (left) and *P. fulvescens* Hadoram Shirihai

A plot of breeding distribution overlap versus clade age showed a general increase in sympatry with increasing time of divergence (Figure 4), with widespread sympatry only after c. 3.7 mya. One major exception concerned *P. fulvescens* and *P. koslowi*, which overlap broadly in Mongolia, despite an estimated divergence of only 2.4 mya (95% HPD 1.8–3.4 mya). The latter is one of only two pairs of sister species with sympatric breeding ranges; the other one is the much older pair *P. collaris*–*P. himalayana* (4.1 mya; 95% HPD 2.6–5.8 mya).

**Figure 5:**
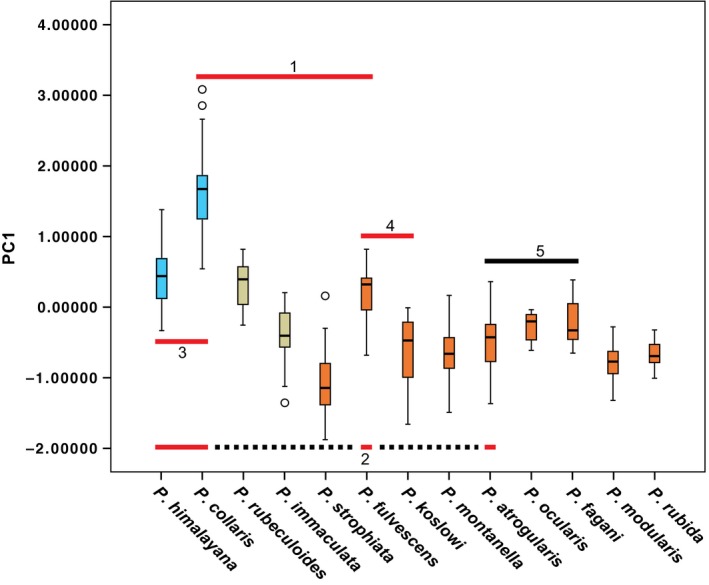
Box plot of PC1 from principal component analysis of morphometrics, demonstrating generally pronounced structural divergence among sympatric species. Red bar 1—five widely sympatric species in the eastern Sino‐Himalayan Mountains; red bar 2—four widely sympatric species in Central Asia (dotted line connects the species that are not adjacent in the figure); red bar 3 and 4—two widely sympatric species pairs; number 4 represents the most recently diverged sympatric species pair; black bar 5—clade E in Figures 2, 3, 4 (all allopatric). Species with pale blue boxes are in clade B, and species with orange boxes are in clade C

In the PCA of morphometrics, one principal component had eigenvalue >1, and explained 65.4% of the variance, with the following contributions: wing 0.940, tail 0.514, bill length 0.902. A boxplot of PC1 scores revealed slight or no overlap in PC scores among most species with widely sympatric breeding ranges, but much broader overlap in PC scores among most of the mainly or entirely allo‐/parapatric species in clade C (Figure 5, Table S6). Of the sympatric species, only *P. rubeculoides* and *P. fulvescens* within the group labeled “1” in Figure 5, and *P. himalayana* and *P. fulvescens* within group “2,” have widely overlapping, nonsignificantly different, PC scores, whereas the other sympatric species have significantly different PC scores (Table S6). In contrast, most of the species in clade C have broadly overlapping, nonsignificantly different, PC scores (Table S6).

On overall size, as gauged by wing length, the species fall into two major groups (Figure S8) corresponding to clades A and B.

## 4 DISCUSSION

### 4.1 Phylogeny

We consider the best estimate of the phylogeny to be compatible with the *BEAST and concat18p trees when all poorly supported clades have been collapsed, that is with six lineages in clade C (containing in total nine species) forming a polytomy (indicated by § in Figures 2 and 3). In contrast, the concat12p tree is fully resolved, with all except one node having PP ≥.95. However, in this tree, three of the internal nodes within clade C have MLBS <50%. All nodes with low MLBS support are extremely short, suggesting that MrBayes might have assigned spuriously high support to these (e.g., Lewis, Holder, & Holsinger, 2005). The *P. montanella* + *P. rubida* clade, which receives high PP and moderate MLBS support in the concat12p and unpartitioned trees, is strongly contradicted by the concatenated nuclear tree, and accordingly probably derives mainly or exclusively from the mitochondrial data. Drovetski et al.'s (2013) ND2 + ACO1 tree inferred *P. montanella* and *P. rubida* as sisters with PP .97. The same clade was found with PP 1.00 in their ND2 tree, but not in their ACO1 tree, again suggesting that this was based exclusively on mtDNA, and for that reason requires corroboration. The Bayes Factor analysis strongly favored the concat 18p over the other partition schemes, lending further support to a more poorly resolved tree over a well resolved one.

Despite analysis of 11 unlinked loci, clade C is largely unresolved in the *BEAST and concat18p trees. The most likely explanation for the lack of resolution within clade C is that six lineages separated very close in time, as suggested by the chronogram and by the short internodes in the multilocus analyzes, and by the network‐like pattern at the base of clade C in the SplitsTree4 tree. This probably resulted in insufficient imprinting of phylogenetic signal in these loci or/and conflicting gene trees caused by stochastic lineage sorting (e.g., Avise & Robinson, 2008; Rokas & Carroll, 2006; Suh, 2016). If the poor resolution among the six major lineages in clade C reflects a hard or near‐hard polytomy, as we suggest might be the case, a more fully resolved phylogeny will be difficult or impossible to obtain by adding more data (e.g., Rokas & Carroll, 2006; Suh, 2016).

The phylogeny is congruent with the groups defined based on plumage similarity by Hatchwell (2005) only with respect to *P. collaris* + *P. himalayana*. Two other groups are incompatible with the phylogeny: (1) *P. modularis* + *P. rubida* + *P. immaculata*, and (2) *P. montanella* + *P. fulvescens* + *P. koslowi* + *P. atrogularis* + *P. ocularis* + *P. fagani*. Both *P. rubeculoides* and *P. strophiata* were considered to have no close affinities by Hatchwell (2005). However, all species with a prominent pale supercilium and contrasting dark crown and ear‐coverts are in clade C, and, in agreement with Hatchwell (2005), the closely similar *P. atrogularis*,* P. ocularis*, and *P. fagani* form a strongly supported clade (E). The unresolved relationships within clade C precludes evaluation of whether the plumage similarity between the geographically widely separated *P. modularis* and *P. rubida* is due to convergence (see below).

### 4.2 Biogeography

Species representing the deep branches of the phylogeny occur in the mountain ranges of Central Asia–Mongolia and the Sino‐Himalayan Mountains. One of these (*P. collaris*) extends its range across all the higher mountains westward to southern Europe and North Africa and eastward to southeast Russia, Japan and Taiwan of China—a most unusual distribution for a bird. The RASP analysis suggests an origin in the Sino‐Himalayan Mountains or this area plus Central Asia–Mongolia. However, because of the somewhat uncertain position of the accentors in relation to other passerine bird families (cf. Alström et al., 2014; Johansson, Fjeldså, & Bowie, 2008), the precise ancestral area of *Prunella* remains uncertain.

The overlapping distributions of *P. collaris*,* P. immaculata*, and *P. rubeculoides* blur the traces of the early speciation events within the ancestral area. Age, connectivity of mountain ranges, and monsoon systems that maintained a favorable climatic impact from the Indian Ocean over millions of years (Fjeldså, Bowie, & Rahbek, 2012) may have allowed these species lineages to disperse and persist across large parts of these mountain regions. Only *P. collaris* has expanded out of this region, giving rise to genetically divergent populations.

The early divergences along the branch leading to clade C were inferred to have taken place within the family's ancestral area, at rather long‐time intervals during the late Miocene–early Pliocene. This was followed by rapid divergence of the six primary lineages in clade C during the mid‐Pliocene to early Pleistocene. The chronogram suggests, albeit with large confidence intervals, that these six primary lineages with uncertain interrelationships diverged within two million years. While *P. strophiata* in clade C occurs within the family's ancestral area, the five other primary lineages in this clade are mainly or wholly allopatric and spread out across the temperate parts of Eurasia. It is not possible to determine with certainty whether the extremely similar‐looking *P. modularis* and *P. rubida*, which inhabit the westernmost and easternmost parts of the geographical range of the genus, respectively, are closely related or not. A sister relationship would suggest that their most recent common ancestor was once much more widespread (cf. such disjunct west‐east distributional patterns in *Cyanopica* magpies (Zhang et al., 2012), *Sitta* nuthatches (Pasquet et al., 2014), and shrikes *Lanius* (Olsson, Alström, Svensson, Aliabadian, & Sundberg, 2010)), whereas a more distant relationship would indicate plumage convergence.

Our biogeographical reconstructions differ from the one by Drovetski et al. (2013), especially with respect to clade C. Drovetski et al. (2013) concluded that the initial split within clade C was into a mainly Western Palearctic (*P. modularis*,* P. atrogularis*,* P. ocularis*,* P. fagani*) and a Central/Eastern Palearctic clade (*P. rubida*,* P. montanella*,* P. strophiata*,* P. fulvescens*,* P. koslowi*). This would seem reasonable from a biogeographical perspective, but as this topology was not recovered in any of our analyzes, and lacks statistical support in Drovetski et al.'s (2013) tree, we consider that scenario to be hypothetical. Moreover, there is strong disagreement in inferred dates between the present study and Drovetski et al.'s (2013). The discrepancy becomes exaggerated toward the present (e.g., split between clades A and B 7.31 mya according to Drovetski et al. vs. 9.1 mya in the present study; initial split within clade C 2.1 mya vs. 4.4 mya; *P. fulvescens*–*P. koslowi* 0.91 mya vs. 2.4 mya). The divergence times in Drovetski et al. (2013) were calculated using “the mean rate of sequence evolution and associated 95% confidence interval,” derived from Hawaiian honeycreepers (Drepanidinae), and then estimating the rate of ACO1 relative to that of ND2. In contrast, the present study used the well‐established average rate of cyt*b* evolution (Weir & Schluter, 2008). Unless the 2.1%/my molecular clock rate for cyt*b* is highly erroneous in *Prunella*, the dates presented by Drovetski et al. (2013) are hard to reconcile with genetic distances; for example, the 0.91 mya split between *P. fulvescens* and *P. koslowi*, which have an uncorrected cyt*b* difference of 4.6%–4.7%. Moreover, Drovetski et al. (2013) included five highly divergent outgroup genera in their dating analysis, whereas we only analyzed the ingroup.

It is interesting to compare the inferred *Prunella* divergence times to those estimated for two unrelated groups of passerines with largely similar distributions and habitats, the *Carpodacus* rosefinches and the snowfinches (genera *Montifingilla*,* Pyrgilauda*, and *Onychostruthus*; unlike *Prunella*, no boreal species, and all occurring in barren habitats). A recent analysis (Tietze, Päckert, Martens, Lehmann, & Sun, 2013) suggested that the deepest split within *Carpodacus* occurred at 14.19 mya (95% HPD 11.68–17.28 mya), and that there was much speciation from c. 10.5 to c. 4.5 mya, that is during a slow‐down in *Prunella* diversification, but also particularly high diversification rates during the main radiation of the *Prunella* clade C (c. 4.5–2 mya). The snowfinch divergence times, between c. 1.5 and 2.5 mya, were only roughly estimated based on cyt*b* distances and a 2% divergence/my. As they were based on uncorrected distances, they are likely to be underestimated, although they are probably within the same time period as the split between *P. fulvescens* and *P. koslowi*.

### 4.3 Dispersal dynamics and contemporary distributions

The rapid divergence and expansion of multiple lineages suggest some common causal factor. As most of the divergences are pre‐Pleistocene, the Pleistocene glaciations were not a major driving force. However, the major climatic oscillations during the Pleistocene may have modified the distributions. As most accentors are adapted to scrubby or barren habitats at high elevations, glacial periods should not have prevented them from breeding north of the Qinghai‐Tibet Plateau, and they may even have bred in the shrub steppe (“mammoth steppe”) of Siberia (see Allen et al., 2011 for ecological conditions). Accordingly, colonizations of new areas were facilitated during glacial periods, when suitable accentor habitats expanded west‐, east‐, and northward from the Qinghai‐Tibet Plateau and the great Asian mountain ranges across intervening low‐elevation areas (e.g., Allen et al., 2011), followed by fragmentation of the suitable habitats during interglacial periods. This is supported by multiple late Pleistocene fossils of *P. collaris* from the lowlands between mountainous regions in Europe (Tyrberg, 1991). Such expansion and subsequent isolation could potentially explain the distributions of at least *P. ocularis*,* P. fagani*,* P. modularis*, and *P. rubida*, as well as the patchy distributions of *P. collaris*,* P. montanella*, and *P. atrogularis*. Although this idea has been proposed by Tyrberg (1991), it has not been widely appreciated, and is contrary to the usual view that glacial periods lead mainly to fragmentation of populations (e.g., Qu et al., 2012; Weir & Schluter, 2004; Zhao et al., 2012).

An alternative hypothesis for the distributions of the isolated *P. fagani* in Yemen and *P. collaris fennelli* in Taiwan of China, or even *P. rubida* in Japan, is that they originated from permanent settlement by migratory ancestors in former winter quarters, as has been suggested for other species (e.g., Escalante, Márquez‐Valdelamar, De La Torre, Laclette, & Klicka, 2009; Voelker & Light, 2011; Winger, Barker, & Ree, 2014). *Prunella* species are mostly resident today, except for the wholly migratory northerly breeding *P. montanella* and the northern populations of *P. modularis* and *P. atrogularis* (Hatchwell, 2005; Snow & Perrins, 1998) and the mainly migratory *P. himalayana* (Ayé, Schweizer, & Roth, 2012; Rasmussen & Anderton, 2012; *contra* Hatchwell, 2005). However, the most widespread species, *P. collaris* apparently has the potential for long‐distance dispersal, as stray individuals have been observed far away from the nearest breeding areas, for example in the UK and Sweden (Snow & Perrins, 1998). Our data do not allow testing of this hypothesis.

The establishment of northern migratory populations could be the result of northward population expansion as new habitats became available during the Holocene. However, it could also be a recurrent phenomenon during warmer climatic episodes (review in Zink, 2011), and it is possible that some populations may have remained there for long periods. It is important to note that Asia differs greatly from Europe in terms of Pleistocene dynamics, as most of Russia east of the great Scandinavian ice sheet remained ice‐free during the Pleistocene glacial cycles (Svendsen et al., 2004). Palynological evidence supports potential *Prunella* habitat throughout the last glacial maximum in many parts of Siberia (Allen et al., 2011). Much denser geographical sampling would be required to evaluate whether the migratory northern populations of *P. atrogularis*,* P. montanella*, and *P. modularis* were established through recent (Holocene) expansion and gain of migratory habits, or if northern populations were maintained through the Pleistocene.

### 4.4 Evolution of communities of sympatric species

With one exception, species in clades with an inferred age of less than c. 3.7 my are allopatric nonsister species. The single exception is the sister pair *P. fulvescens*–*P. koslowi*, which is widely sympatric in Mongolia, with a divergence time of 2.4 mya (95% HPD 1.5–3.4 mya). Only one other sister pair breeds sympatrically, the much more anciently diverged (4.1 mya, 95% HPD 2.6–5.8 mya) *P. collaris–P. himalayana*. The distributional pattern and inferred ages suggest speciation in geographically separate areas and a substantial waiting time until secondary contact. Thus, the first species to become established in an area may exclude colonization by other closely related species. This has been suggested to be the main factor limiting speciation in Himalayan passerines (Price et al., 2014) and the Neotropical avian family Furnariidae (Pigot & Tobias, 2013). A significant divergence in allopatry appears to be needed before species can coexist in sympatry (e.g., Pigot & Tobias, 2013; Price & Kirkpatrick, 2009; Price et al., 2014; Webb et al., 2002). Drovetski et al. (2013) reached similar conclusions regarding the allopatric mode of speciation in *Prunella*, although, as already noted, their dates were much younger: allopatry for all lineages younger than 1.5 my, although only 0.91 mya (95% HPD 0.55–1.29 mya) for *P. fulvescens* and *P. koslowi*. Our divergence times to sympatry are more in line with earlier studies of various groups of birds (e.g., Price et al., 2014).

All sympatrically breeding species are ecologically segregated, as suggested by differences in size/structure and habitat. The PCA plot suggests that widely sympatric species are generally more divergent in size and/or structure than the allo‐/parapatric ones. This could be a simple reflection of the longer times of separation of most of the sympatric species. However, also the most recently diverged sympatric species pair, *P. fulvescens*–*P. koslowi*, is well separated in the PCA plot, supporting the importance of biotic interactions in limiting or allowing co‐existence (as also suggested by other studies, e.g., Pigot & Tobias, 2013; Price et al., 2014). In fact, the widely sympatric *P. fulvescens* and *P. strophiata* are the most divergent species within the comparatively recently radiated clade C. Although it is possible that divergence in size and structure could have been enforced during secondary contact, it seems more likely that only species that have developed sufficient differences in these respects, and hence ecology, are able to coexist (see also plumage, below). The early separation into two major groups based on size (clades A and B) agrees with the results from an analysis of the entire Himalayan passerine radiation, where body size and shape differences evolved before differences in elevational distributions (Price et al., 2014).

The five sympatric species in the Sino‐Himalayan Mountains have more or less divergent habitat preferences: *P. collaris* in stony, sparsely vegetated alpine habitats, generally higher than the others; *P. rubeculoides* in subalpine scrubby, often somewhat wet, habitats, generally ≥4,000 m a.s.l.; *P. fulvescens* in subalpine/alpine dry, barren, rocky and sparsely scrubby habitats, at similar elevation as *P. rubeculoides*;* P. strophiata* in dense scrub above the treelimit and in open coniferous forest near the upper tree limit, generally below *P. rubeculoides* and *P. fulvescens*; and *P. immaculata* in moist coniferous forest with rhododendron, mainly below *P. strophiata* (Hatchwell, 2005; Rasmussen & Anderton, 2012; Portenko & Vietinghoff‐Scheel, 1977; Vietinghoff‐Scheel, 1974, 1977; Per Alström pers. obs.). However, the differences are not clear‐cut, and at least *P. rubeculoides*,* P. fulvescens* and *P. strophiata* may breed alongside each other in alpine habitats with very low scrub in central China (Per Alström pers. obs.). All of these, except *P. rubeculoides* and *P. fulvescens*, are clearly segregated in morphospace (cf. Figure 5).

Also the four species breeding in sympatry in Central Asia differ in habitat choice. The sisters *P. collaris* and *P. himalayana* are extensively sympatric in alpine habitats, though the former breeds on average higher (Ayé et al., 2012; Hatchwell, 2005; Vietinghoff‐Scheel, 1974). *Prunella fulvescens* breeds on average lower than both previous species, in alpine and subalpine habitats and scrub, whereas *P. atrogularis* occurs in dense scrub above the tree limit and in forest (Ayé et al., 2012; Hatchwell, 2005; Per Alström pers. obs.). All of these, except *P. himalayana* and *P. fulvescens*, are clearly segregated in morphospace (cf. Figure 5).

The most recently separated (2.4 mya; 95% HPD 1.5–3.4 mya) widely sympatric species, *P. fulvescens* and *P. koslowi*, have at least partly different habitat preferences, the former favouring dry, rocky, sparsely scrubby habitats at high elevation, whereas the latter prefers scrub in mountain valleys and slopes or semidesert at on average lower altitude (Gombobaatar et al., 2011; Hatchwell, 2005; Vietinghoff‐Scheel, 1971; Per Alström pers. obs.). However, at least in the Gobi Altai they do breed next to each other in extensive patches of *Juniperus* around c. 2,400 m a.s.l. (Per Alström pers. obs.). Their coexistence may be facilitated by marked differences in size/structure as well as plumage (see below).

*Prunella modularis* has an extraordinarily wide habitat range, breeding in the presumably ancestral scrubby high elevation habitat of clade C in the mountains of western Asia and south and central Europe, but descending to the lowlands in other parts, and even breeds commonly in forests, gardens and parks down to sea level in western Europe (Hatchwell, 2005; Snow & Perrins, 1998). A parallel biogeographical pattern is shown in the avian family Cettiidae. All except one of the continental Asian species and single African representative occur in mountains, from foothills to above the tree line, whereas the single Western Palearctic species, *Cettia cetti*, is found in the plains (in wet habitats, unlike all its relatives) (cf. Kennerley & Pearson, 2010). *Prunella collaris* inhabits an even wider elevational span than *P. modularis*, and in fact has the broadest elevational distribution of any bird in the world, from close to 8,000 m (commonly to at least 5,600 m) in the Himalayas down to sea level in coastal southeast Russia (Hatchwell, 2005). Its typical alpine habitat is rather closely matched by the tundra habitat along the coast of southeast Russia, highlighting the importance of habitat rather than altitude.

All sympatric species are markedly different in plumage, although the widely sympatric *P. collaris* and *P. himalayana* are overall rather similar. The species with the most similar plumages (*P. modularis*,* P. rubida*;* P. atrogularis*,* P. ocularis*,* P. fagani* [clade E]) are allopatric. The very similar‐looking *P. modularis* and *P. rubida* have widely disjunct distributions, and are not inferred to be closely related, although this is poorly supported. A distant relationship between them could imply remarkable parallel plumage evolution, or retention of an ancestral plumage type. As *P. ocularis* and *P. fagani* are the most recently separated sister species, the lack of strong plumage divergence is not unexpected. However, the two species in the second youngest species pair, *P. fulvescens–P. koslowi*, have broadly overlapping distributions, and are markedly different in plumage. This might indicate selection for signal divergence (reproductive character displacement; Dobzhansky, 1940; Howard, 1993; Liou & Price, 1994; Servedio & Sætre, 2003), or that coincidental plumage divergence in allopatry after separation from the most recent common ancestor facilitated establishment of sympatric breeding ranges.

### 4.5 Taxonomic remarks

The deep divergence between *P. collaris* from the Western Palearctic (*P. c. collaris* and *P. c. montana*) and central China (*P. c. nipalensis*) + Taiwan of China (*P. c. fennelli*), which is on a par with the splits between the two sister pairs *P. ocularis*–*P. fagani* and *P. fulvescens*–*P. koslowi*, suggests that these populations have reached far in the speciation process. Drovetski et al. (2013) found a similarly deep split between *P. c. montana* and *P. c. erythropygia* (latter from Altai mountains to Japan). As suggested by Drovetski et al. (2013), a taxonomic revision of *P. collaris* is called for, although considerably better geographical sampling is required. Also the widely distributed *P. fulvescens*, which shows indications of deep divergences in the present study, should be investigated further.

## 5 CONCLUSIONS

The favored phylogeny has a polytomy comprising six primary lineages, which are considered to have radiated within c. 2 million years during the mid‐Pliocene to beginning of Pleistocene. Other, more resolved trees based on different partition schemes, are for various reasons considered less trustworthy, despite overall higher posterior probabilities. These results emphasize the importance of carefully evaluating results even when support values are high. We conclude that the lack of resolution at the base of clade C is most likely due to the rapid radiation, and might prove to be a “hard polytomy.”

We conclude that divergences took place in allopatry, with long waiting times before establishment of sympatry, and a general increase in sympatry with increasing time since divergence. Only two pairs of sister species are sympatric, and all except one of the clades that include sympatric species are older than c. 3.7 my. All sympatrically breeding species are ecologically segregated, as suggested by differences in size/structure and habitat choice. All sympatric species, including the most recently diverged sister pair *P. fulvescens–P. koslowi* (2.4 mya), also differ markedly in plumage. Although it is possible that divergence in size/structure and plumage could have been enforced during secondary contact, we suggest that only species that differ sufficiently in these respects, and hence ecology, are able to coexist.

Colonizations of new areas were facilitated during glacial periods, when suitable habitats expanded across lowlands, followed by fragmentation during interglacials. This scenario is contrary to the usual view that glacial periods result mainly in fragmentation.

## CONFLICT OF INTEREST

None declared.

## AUTHOR CONTRIBUTIONS

The study was designed by P.A. and F.L. DNA samples were provided by U.O., J.F., T.S., C.‐t.Y., Y.Q., and F.L. Distributional data were provided by Q.Q. and C.S.R. Morphological data were provided by C.S.R. and P.A. Laboratory work was carried out by B.L., U.O., and Y.H. All analyzes were carried out by P.A. and B.L. The first draft was written by P.A. and B.L. All authors provided input to various versions of the manuscript, and approved of the final version.
