# Supplementary material for: Explosive radiation and spatial expansion across the cold environments of the Old World in an avian family
Source: Ecol Evol. 2017 Jul 6;7(16):6346–57. doi: 10.1002/ece3.3136 (PMC5574758; doi:10.1002/ece3.3136)
Supplement: Supplementary file 1 [file ECE3-7-6346-s001.docx]

**APPENDIX S1**

**MATERIAL AND METHODS**

**DNA extraction and sequencing**

For the *Prunella* and four outgroup species (*Aethopyga christinae*, *Dicaeum cruentatum*, *Passer montanus* and *Chloropsis hardwickii*), we sequenced two mitochondrial genes, three Z-linked loci and seven autosomal loci. The mitochondrial genes were cytochrome c oxidase I (COI) and cytochrome *b* (cyt*b*); the Z-linked introns were chromo-helicase-DNA binding protein intron 1 (CHD1Z), brama protein intron (BRM) and Z6; and the nuclear loci were recombination-activating gene (RAG1), oxoglutarate dehydrogenase-like protein gene intron (OGDHL), transforming growth factor β intron 2 (TGFB), myoglobin intron 2 (myo), ornithine decarboxylase (mainly) introns 6–7 (ODC), glyceraldehyde-3-phosphodehydrogenase intron 11 (GAPDH), and an anonymous locus (Loc19; Gao *et al*., 2012). Information about the primers are given in Table S2). All new sequences have been deposited in GenBank (Table S1). Choice of outgroups was based on e.g. Johansson et al. (2008) and Alström et al. (2014).

**Phylogeny**

Sequences were aligned using Muscle (Edgar, 2004) in Seaview 4.3.4 (Gouy *et al*., 2010; Gouy, 2012); some manual adjustment was done for the non-coding sequences. For the nuclear loci, heterozygous sites were coded as ambiguous. Trees were estimated by Bayesian inference (BI) using MrBayes 3.2 (Huelsenbeck & Ronquist, 2001; Ronquist & Huelsenbeck, 2003) using different data partitioning schemes: (1) all loci were analysed separately (single-locus analyses, SLAs); (2) sequences were concatenated and partitioned by (a) locus (in total 12 partitions) or (b) locus and, for the coding sequences, codon (in total 18 partitions), using rate multipliers to allow different rates for different partitions (Ronquist & Huelsenbeck, 2003; Nylander *et al*., 2004), or (c) unpartitioned. All analyses were run under the best-fit models according to the Bayesian Information Criterion (BIC), calculated in jModeltest 0.1.1 (Posada, 2008a, b), as well as using the ‘mixed’ command to sample across the GTR model space in the Bayesian MCMC (Huelsenbeck *et al*. 2004), and (a) assuming rate variation across sites according to a discrete gamma distribution with four rate categories (Γ; Yang, 1994) and an estimated proportion of invariant sites (I; Gu *et al*., 1995) or (b) assuming no rate variation (i.e. just ‘mixed’). We also ran an 18-partition analysis based on the models selected by the BIC in PartitionFinder 1.1.1 (Lanfear et al., 2012).

The following models were selected by jModeltest: cyt*b* and COI, the HKY model (Hasegawa et al., 1985) + Γ + I; CHD1Z, GAPDH, ODC, OGDHL, RAG1 and TGFB, HKY + Γ; myo the general time-reversible (GTR) model (Lanave *et al*., 1984; Rodríguez *et al*., 1990; Tavaré, 1986) + Γ; BRM and Z6, GTR; and Loc19, HKY. The following models were selected by PartitionFinder: BRM, CHD1Z, ODC, OGDHL, GTR + Γ; COI pos 1, RAG1 pos 1, GTR + I; cyt*b* pos 1, cyt*b* pos 3, COI pos 3, GAPDH, the HKY model (Hasegawa et al., 1985) + Γ + I; myo, RAG1 pos 2, RAG1 pos 3, TGFB, Loc19, HKY + Γ; cyt*b* pos 2, COI pos 2, Z6, HKY + I; RAG1 pos 1, HKY. Ambiguous base pairs and indels were treated as missing data, but indels were plotted on the trees *a posteriori*. Default priors in MrBayes were used. Four Metropolis-coupled MCMC chains with incremental heating temperature 0.1 or 0.05 were run for 15–30×10^6^ generations and sampled every 1000 generations. Convergence to the stationary distribution of the single chains was inspected in Tracer 1.5.0 (Rambaut & Drummond, 2009) using a minimum threshold for the effective sample size. The effective sample sizes for the joint likelihood and other parameter values were >1000, representing good mixing of the MCMC. We also examined convergence and reproducibility by running each analysis at least twice, with random starting points. Topological convergence was examined by eye and by the average standard deviation of split frequencies (≤0.01). The first 25% of generations were discarded as ‘burn-in’, and the posterior probabilities (PPs) were calculated from the remaining samples (pooled from the two simultaneous runs).

In order to estimate divergence times, the cyt*b* data set with multiple subspecies was analysed in BEAST version 1.8.2 (Drummond *et al*. 2012). Analyses were run under the GTR + Γ model (cf. Weir & Schluter, 2008) with a ‘birth-death incomplete sampling’ prior. An uncorrelated lognormal relaxed clock (Drummond *et al*., 2006) with a mean clock rate of 2.1%/my (Weir & Schluter, 2008) was applied. Other priors were used with default values, or with a normal distribution on the birth death mean growth rate prior, with initial value 1.0, mean 2.0 and standard deviation 1.0. For these analyses, 200×10^6^ generations were run, sampled every 1000 generations. Every analysis was run twice. As these analyses inferred *P. immaculata* and *P. rubeculoides* to be sisters, with poor support, in conflict with the results from the 18-partion multilocus and *BEAST analyses (see below and Results), we also ran analyses with the topology constrained to match the well supported clades in the 18-partion and *BEAST trees. The MCMC output was analysed in Tracer version 1.5.0 in the BEAST package to evaluate whether valid estimates of the posterior distribution of the parameters had been obtained. The first 50% of the generations were discarded as ‘burn-in’, well after stationarity of chain likelihood values had been established. Trees were summarized using TreeAnnotator version 1.7.4 in the BEAST package, choosing ‘Maximum clade credibility tree’ and ‘Mean heights’, and displayed in FigTree version 1.4.0 (Rambaut, 2012).

To establish how well each model fit the data, we calculated Bayes Factors (BF; Newton & Raftery, 1994; Kass & Raftery, 1995) using the harmonic mean as an approximation of the marginal likelihood of a model.

Integrative species tree estimation was performed using *BEAST (Heled & Drummond, 2010) in the BEAST package, with gene trees and species trees estimated simultaneously. Species delimitation has to be defined *a priori* in *BEAST. We followed the default settings and recommendations of *BEAST to set up the models. We ran analyses under the same substitution models per partition as in the MrBayes analyses, and an uncorrelated lognormal relaxed clock (Drummond *et al*., 2006). We also ran analyses with the coding sequences partitioned by codon. All analyses were run with the outgroup removed as well as with the same four outgroup species as in the MrBayes analyses (see above). A piecewise linear population size model with a constant root was used as a prior for the multispecies coalescent and a birth-death model (Gernhard, 2008) as prior on divergence times. 150–200×10^6^ generations were run, sampled every 1000 generations.

The concatenated data were analysed by maximum likelihood bootstrapping (MLBS). 1000 replicates were run in RAxML-HPC2 version 8.0.0 (Stamatakis, 2006; Stamatakis *et al*., 2008) on the Cipres portal (Miller *et al*., 2010). The data were partitioned by locus, and GTRCAT was used for both the bootstrapping phase and the final tree inference. The data were also analysed by parsimony bootstrapping (MPBS) in PAUP* (Swofford, 2002): heuristic search strategy, 1000 replicates, starting trees obtained by stepwise addition (random addition sequence, 10 replicates), TBR branch swapping, MulTrees option not in effect (only one tree saved per replicate).

**RESULTS**

**Sequence characteristics**

Sequence data were obtained from three to six individuals, except for *P. ocularis*, for which only one sample was available, and for *P. fagani*, for which only two samples were available (both toepads from museum specimens) (Table S1). For all species, all loci were amplified for at least one individual, except for *P. fagani*, for which only cyt*b* was amplified, *P. ocularis*, for which only five loci were amplified, and *P. himalayana* for which one locus was missing from the individual with the most complete set of data (but combined, all loci were amplified for this species) (Table S1).

There were no stop codons in the mitochondrial sequences that could indicate the amplification of nuclear pseudogenes (Sorensen & Quinn 1998), but some double peaks were noted in the chromatograms of the cyt*b* sequences of *P. atrogularis* 141223 and both *P. fagani*. This *P. atrogularis* sequence was recovered in a clade with the same species, and the two *P. fagani* sequences were in a clade with *P. ocularis* and *P. atrogularis*, which has been suggested based on plumage (Hatchwell, 2005), and also recovered by Drovetski *et al*. (2013). Accordingly, if any pseudogenes were included these would have been of so recent origin that they would not have caused problems for the phylogenetic inferences. However, it seems possible that the sequence divergence between the two *P. fagani* sequences has been exaggerated due to difficulties of obtaining good sequences from these old museum specimens. There were no distinct double peaks in the chromatograms of the COI sequences. Sequence lengths are given in Table S2.

**Single-locus analyses**

The trees based on single-locus analyses (hereafter SLAs) varied in resolution and support (Fig. S1). In the cyt*b* and COI trees, all of the species were monophyletic, but most relationships among species were uncertain. The trees based on nuclear loci were less resolved and supported, and the sequences did not sort entirely according to species in any of them (Fig. S1). OGDHL and loc19 were best resolved (39% and 32% of total number of nodes, respectively), and both had a fairly high proportion of monophyletic species (54.5% and 63.6%, respectively, of species with more than one sample). Other trees varied in resolution and number of monophyletic species, with no correlation between these entities.

The only clade that was consistent in all SLAs was the deepest split, between *P. collaris/P. himalayana* (clade B) and the other species (clade A). The reciprocal monophyly of clades A and B and their sister relationship was supported in seven of the 12 trees. The only strongly supported topological conflicts concerned the position of *P. rubeculoides*: in the cyt*b* tree it was sister to *P. immaculata* with PP 1.00 (same topology in COI tree, but with low support); in the OGDHL tree it was sister to *P. collaris*/*P. himalayana* with PP 0.98; and in the ODC, CHD1Z and loc19 trees it was in a clade with all species except *P. immaculata*, *P. collaris* and *P. himalayana* with PP 1.00.

**Concatenation analyses**

The BI tree based on the concatenated mitochodrial sequences (Fig. S2a) resembled the SLAs of these loci. Five clades with more than one species (A–D) received PP ≥0.95 and high MLBS (≥90%). The tree based on the concatenated nuclear sequences (Fig. S2b) was overall better resolved and better supported than any of the nuclear SLAs. Three of the clades that received high support in the mitochondrial tree (A–C) were unanimously strongly supported also in the nuclear tree, whereas the fourth clade (D) received high PP (0.97) but weak MLBS (66%). Within clade A, *P. immaculata* and *P. rubeculoides* were inferred to be successive sisters to the others, with high support. One clade (F) not recovered in the mitochondrial tree obtained high PP (0.98) but low MLBS (64%).

The tree based on the complete concatenated dataset analysed in 18 partitions (12 loci, three further partitioned by codon, mixed + Γ + I; hereafter concat18p; Fig. S3) had significantly higher likelihood than the trees based on the other partition schemes (Table S4). It was fully resolved with respect to interspecific relationships except for one trichotomy within clade C. Clades A–C were unanimously strongly supported. However, within clade C, only clades D and E were strongly supported, whereas other interspecific relationships had low BI and MLBS support. The tree analysed in 18 partitions using the models proposed by PartitionFinder was virtually identical to the concat18p tree (Fig. S4). Also the tree based on the unpartitioned complete data (Fig. S5) was similar to the concat18p tree, exept that the *P. montanella* + *P. rubida* clade, which received low PP in the concat18p tree, had PP 1.00 and MLBS 79%. In contrast, the tree based on the complete concatenated dataset analysed in 12 locus-specific partitions (hereafter concat12p; Fig. S6) was fully resolved, and all except one of the interspecific nodes had PP ≥0.95 (one PP 0.91). However, three of the nodes with PP 0.91–0.99 had MLBS <50%.

Trees from the output of the concat18p analysis were explored in SplitsTree4 (v. 4.14.4) (Huson & Bryant, 2006, 2016). A network rather than bifurcating consensus topology was found for some nodes within clade C when the threshold was set to ≤0.23, with the uncertainty increasing the lower the threshold (threshold 0.1 shown in Fig. 2).

***BEAST tree**

The tree inferred using a codon model for the coding loci did not improve resolution or support compared to the tree where the codons were linked (not shown).

**Indels**

Three indels supporting clades with two or more species and four supporting single species could be mapped unambiguously on the tree, whereas three others exhibited some conflict with the tree (Fig. S2b). Several other indels were highly variable, without any phylogenetic pattern; some varied even among individuals of the same species.

**References**

Alström, P., Hooper, D.M., Liu, Y., Olsson, U., Mohan, D., Gelang, M., Le Manh, H., Zhao, J., Lei, F. & Price, T.D. (2014) Discovery of a relict lineage and monotypic family of passerine birds. *Biology Letters*, **10**, 20131067.

Drummond, A.J., Ho, S.Y.W., Phillips, M.J. & Rambaut, A. (2006) Relaxed phylogenetics and dating with confidence. *PLoS Biology*, **4**, e88.

Drummond, A.J., Suchard, M.A., Xie, D. & Rambaut, A. (2012) Bayesian phylogenetics with BEAUti and the BEAST 1.7. *Molecular Biology and Evolution*, **29**, 1969–1973.

Edgar, R.C. (2004) MUSCLE: multiple sequence alignment with high accuracy and high throughput. *Nucleic Acids Research*, **32**, 1792–1797.

Gao, B., Qu, Y., Song, G., Liu, H. & Lei, F. (2012) Anonymous single-copy nuclear DNA (scnDNA) markers for Grey-cheeked Fulvetta (*Alcippe morrisonia*) and Rufous-capped Babbler (*Stachyridopsis ruficeps*). *Conservation Genetics Resources*, **4**, 777–781.

Gernhard, T. (2008) The conditioned reconstructed process. *Journal of Theoretical Biology*, 253, 769–778.

Gouy, M. (2012) *Seaview*, Version 4.3.4. Available at: http://pbil.univ-lyon1.fr/software/seaview.html

Gouy, M., Guindon, S. & Gascuel, O. (2010) SeaView version 4: a multiplatform graphical user interface for sequence alignment and phylogenetic tree building. *Molecular Biology and Evolution*, **27**, 221–224.

Gu, X., Fu, Y.-X. & Li, W.-H. (1995) Maximum likelihood estimation of the heterogeneity of substitution rate among nucleotide sites. *Molecular Biology and Evolution*, **12**, 546–557.

Hasegawa, M., Kishino, H. & Yano, T.-a. (1985) Dating of the human-ape splitting by a molecular clock of mitochondrial DNA. *Journal of Molecular Evolution*, **22**, 160–174.

Hatchwell, B.J. (2005) Family Prunellidae (Accentors). *Handbook of the Birds of the World* (ed. by J. Del Hoyo, J. Elliott and D.A. Christie), pp. 496–513. Lynx Edicions,, Barcelona.

Heled, J. & Drummond, A.J. (2010) Bayesian inference of species trees from multilocus data. *Molecular Biology and Evolution*, **27**, 570–580.

Huelsenbeck, J.P. & Ronquist, F. (2001) MRBAYES: Bayesian inference of phylogenetic trees. *Bioinformatics*, **17**, 754–755.

Huelsenbeck, J.P., Larget, B. & Alfaro, M.E. (2004) Bayesian phylogenetic model selection using reversible jump Markov chain Monte Carlo. *Molecular Biology and Evolution*, **21**, 1123-1133.

Johansson, U.S., Fjeldså, J. & Bowie, R.C.K. (2008) Phylogenetic relationships within Passerida (Aves: Passeriformes): a review and a new molecular phylogeny based on three nuclear intron markers. *Molecular Phylogenetics and Evolution*, **48**, 858–876.

Kass, R.E. & Raftery, A.E. (1995) Bayes factors. *Journal of the American Statistical Association*, **90**, 773–795.

Lanave, C., Preparata, G., Sacone, C. & Serio, G. (1984) A new method for calculating evolutionary substitution rates. *Journal of Molecular Evolution*, **20**, 86–93.

Lanfear, R., Calcott, B., Ho, S.Y. & Guindon, S. (2012) PartitionFinder: combined selection of partitioning schemes and substitution models for phylogenetic analyses. *Molecular Biology and Evolution*, **29**, 1695-1701.

Miller, M.A., Pfeiffer, W. & Schwartz, T. (2010) Creating the CIPRES Science Gateway for inference of large phylogenetic trees. *Gateway Computing Environments Workshop* (GCE), 2010 (ed by, pp. 1–8.

Newton, M.A. & Raftery, A.E. (1994) Approximate Bayesian inference with the weighted likelihood bootstrap. *Journal of the Royal Statistical Society*. Series B (Methodological), **56**, 3–48.

Nylander, J.A.A., Ronquist, F., Huelsenbeck, J.P. & Nieves-Aldrey, J. (2004) Bayesian phylogenetic analysis of combined data. *Systematic Biology*, **53**, 47–67.

Posada, D. (2008) jModelTest: phylogenetic model averaging. *Molecular Biology and Evolution*, **25**, 1253-1256.

Posada, D. (2008) *jModeltest*. version 0.1.1. Available at: https://code.google.com/p/jmodeltest2

Rambaut, A. (2012) *FigTree* 1.4.0. Available at: http://tree.bio.ed.ac.uk/software/figtree/ (accessed

Rambaut, A. & Drummond, A.J. (2009) *Tracer*, version 1.5. Available at: http://beast.bio.ed.ac.uk (accessed

Rambaut, A. & Drummond, A.J. (2012) *TreeAnnotator*., version 1.7.4. Available at: http://beast.bio.ed.ac.uk. (accessed

Rodriguez, F., Oliver, J.L., Marin, A. & Medina, J.R. (1990) The general stochastic model of nucleotide substitution. *Journal of Theoretical Biology*, **142**, 485–501.

Ronquist, F. & Huelsenbeck, J.P. (2003) MrBayes 3: Bayesian phylogenetic inference under mixed models. *Bioinformatics*, **19**, 1572–1574.

Sorenson, M.D. & Quinn, T.W. (1998) Numts: a challenge for avian systematics and population biology. *The Auk*, **115**, 214–221.

Stamatakis, A. (2006) RAxML-VI-HPC: maximum likelihood-based phylogenetic analyses with thousands of taxa and mixed models. *Bioinformatics*, **22**, 2688-2690.

Stamatakis, A., Hoover, P. & Rougemont, J. (2008) A rapid bootstrap algorithm for the RAxML Web servers. *Systematic Biology*, **57**, 758–771.

Swofford, D. (2002) *PAUP** 4.0 : Phylogenetic Analysis Using Parsimony. Sinauer Associates.

Tavaré, S. (1986) Some probabilistic and statistical problems in the analysis of DNA sequences. *Lect. Math. Life Sci.*, **17**, 57-86.

Weir, J.T. & Schluter, D. (2008) Calibrating the avian molecular clock. *Molecular Ecology*, **17**, 2321-2328.

Yang, Z. (1994) Maximum likelihood phylogenetic estimation from DNA sequences with variable rates over sites: approximate methods. *Journal of Molecular Evolution*, **39**, 306-314.
