# Supplementary figures and images for: Explosive radiation and spatial expansion across the cold environments of the Old World in an avian family
Source: Ecol Evol. 2017 Jul 6;7(16):6346–57. doi: 10.1002/ece3.3136 (PMC5574758; doi:10.1002/ece3.3136)

Cytochrome b

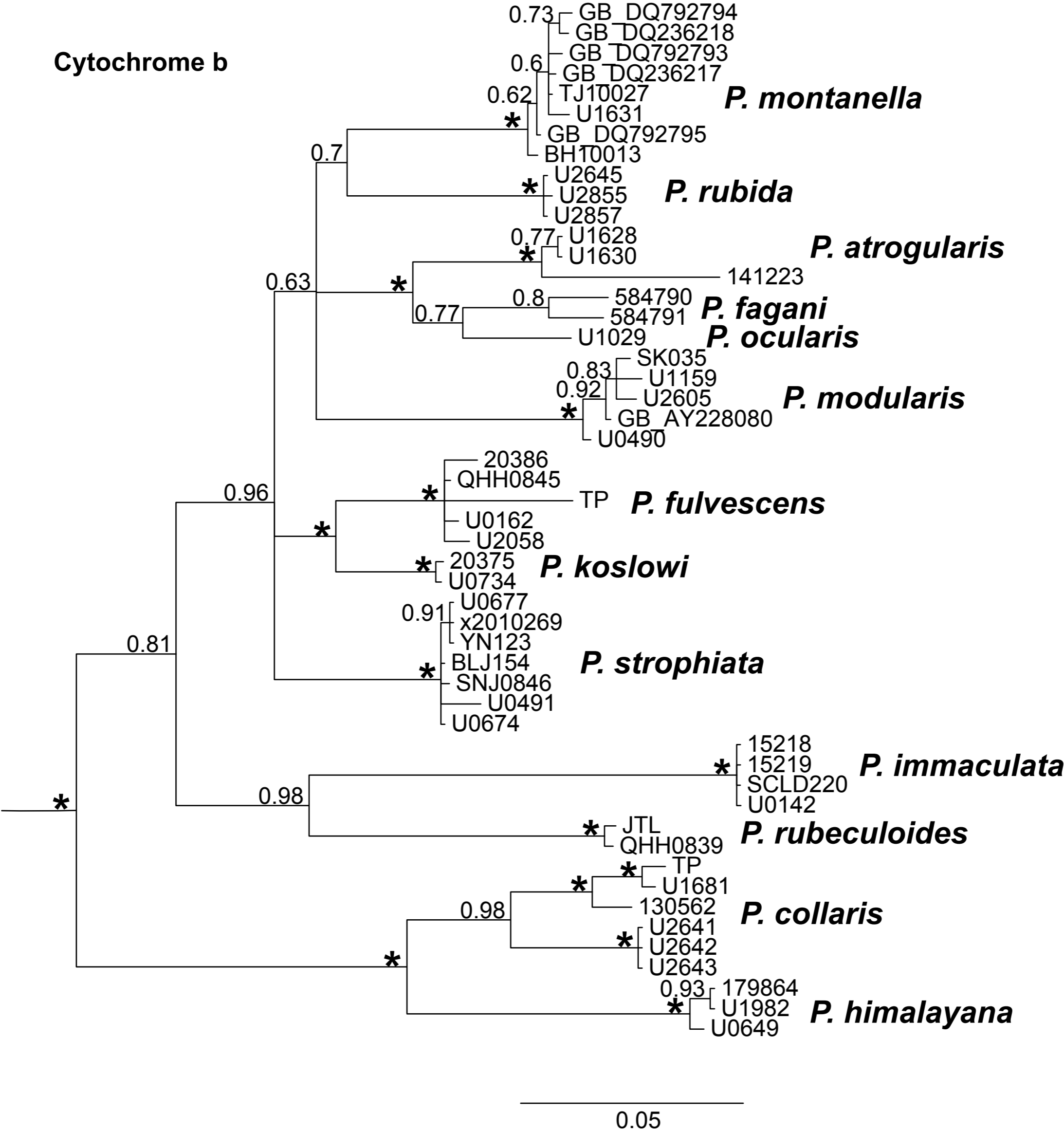

CO1

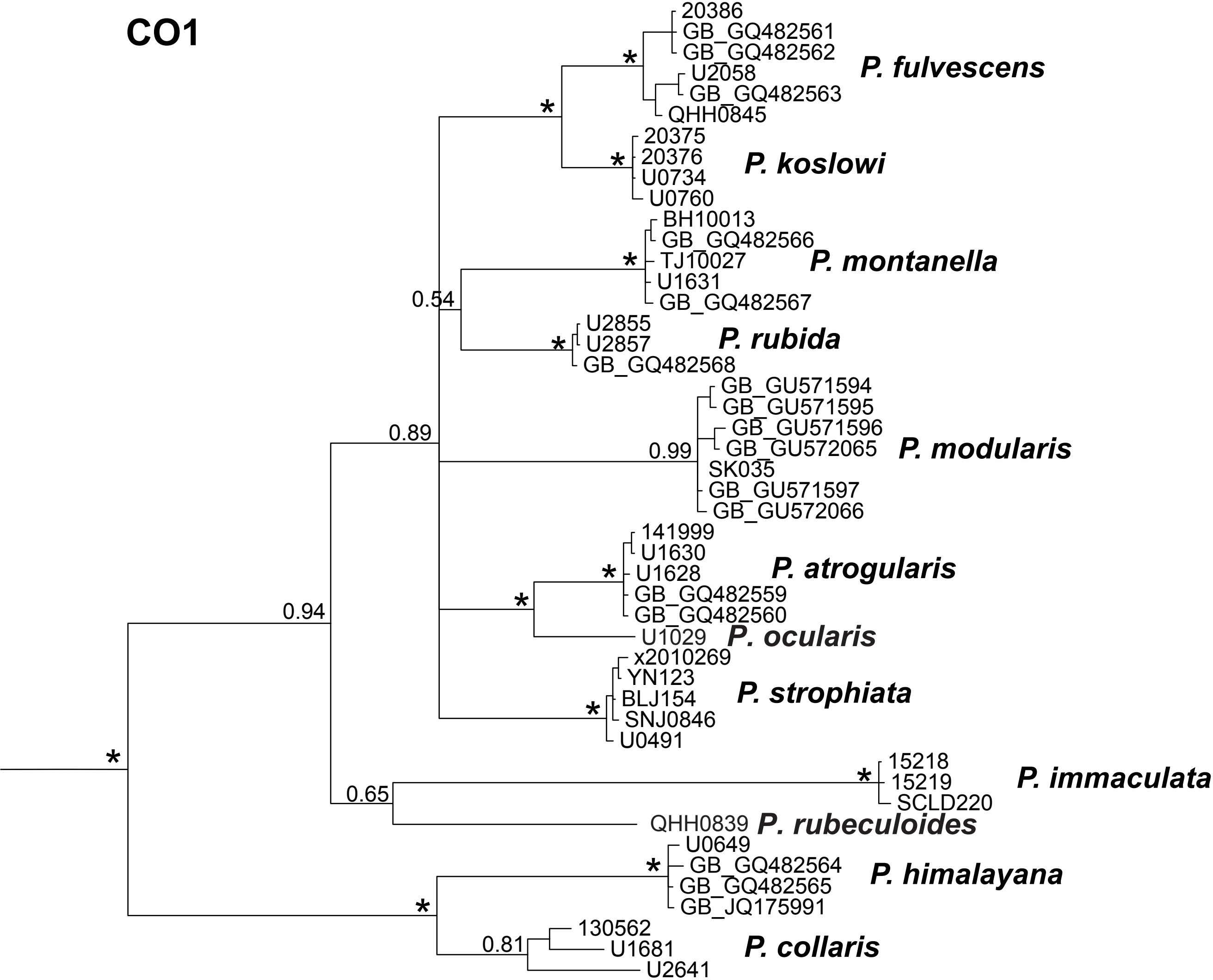

0.05

GAPDH

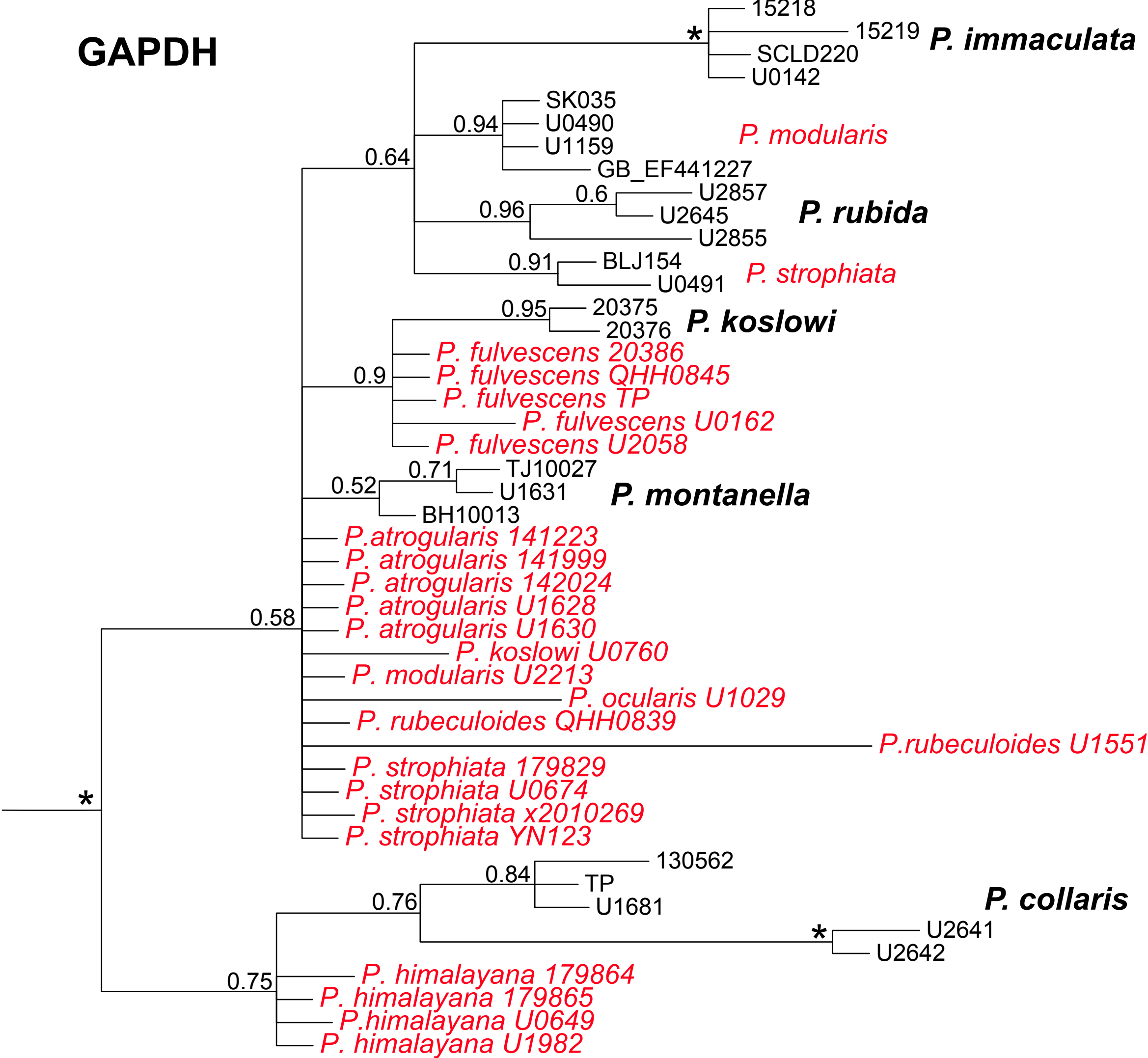

Myo

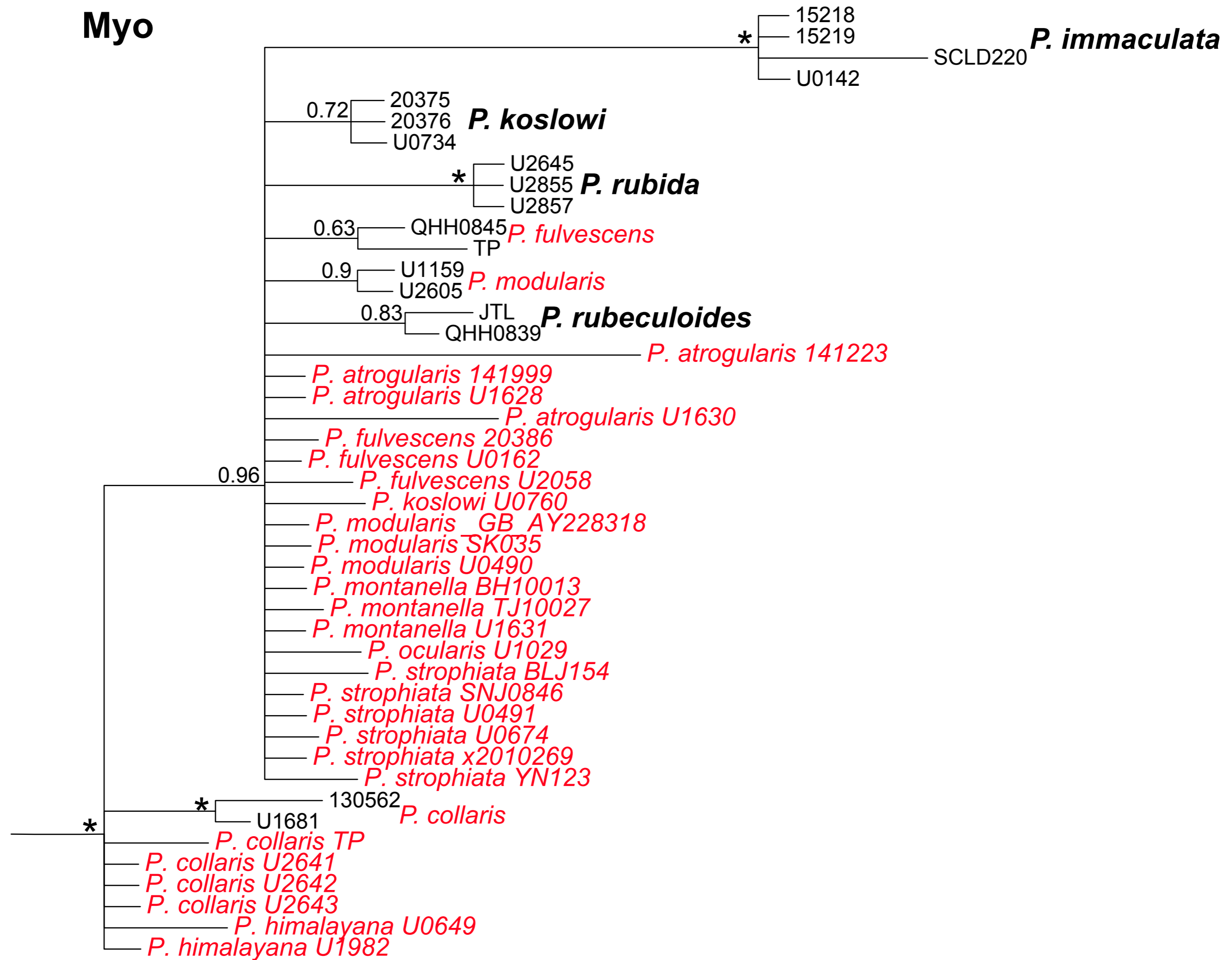

ODC

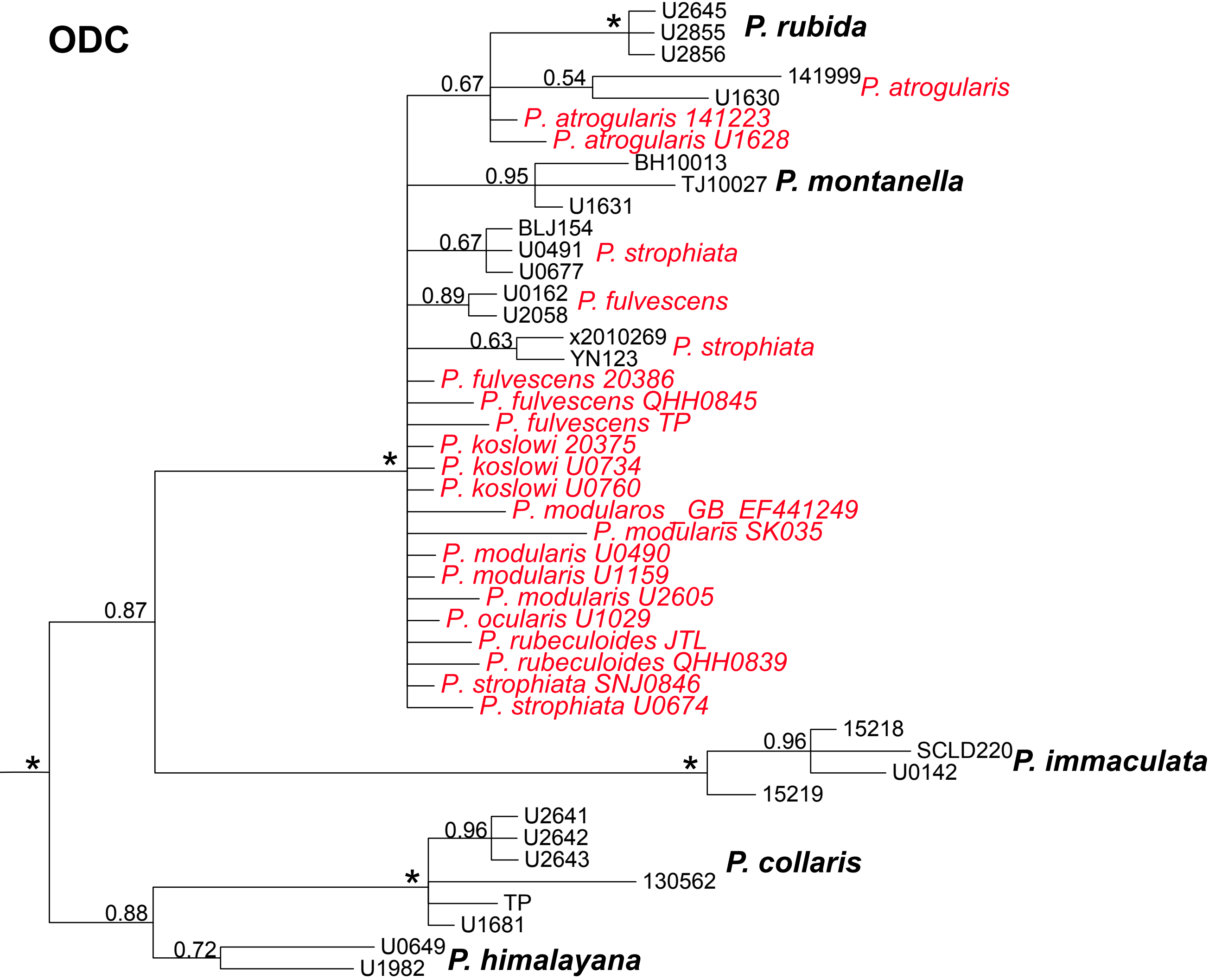

OGDHLF

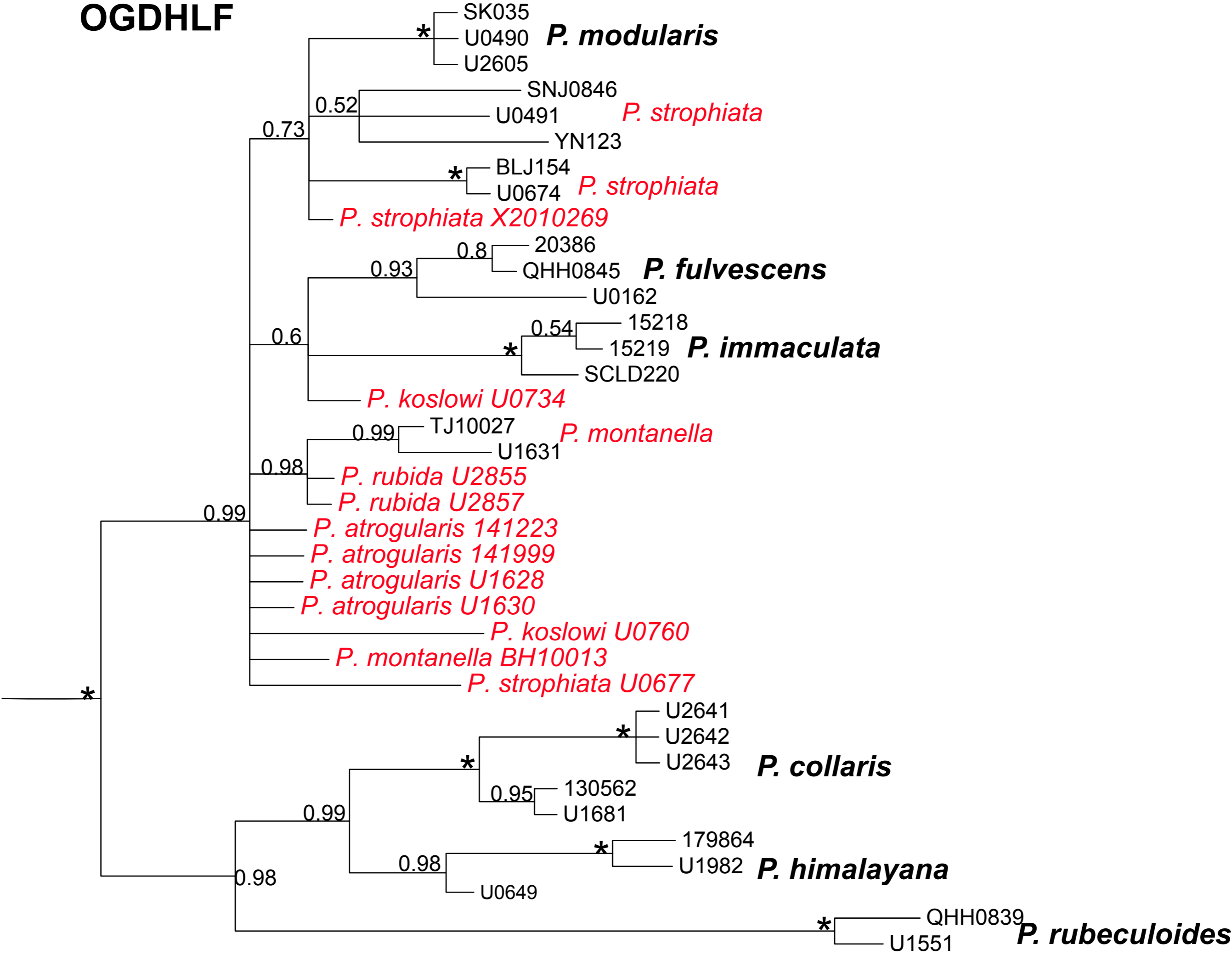

0.008

RAG1

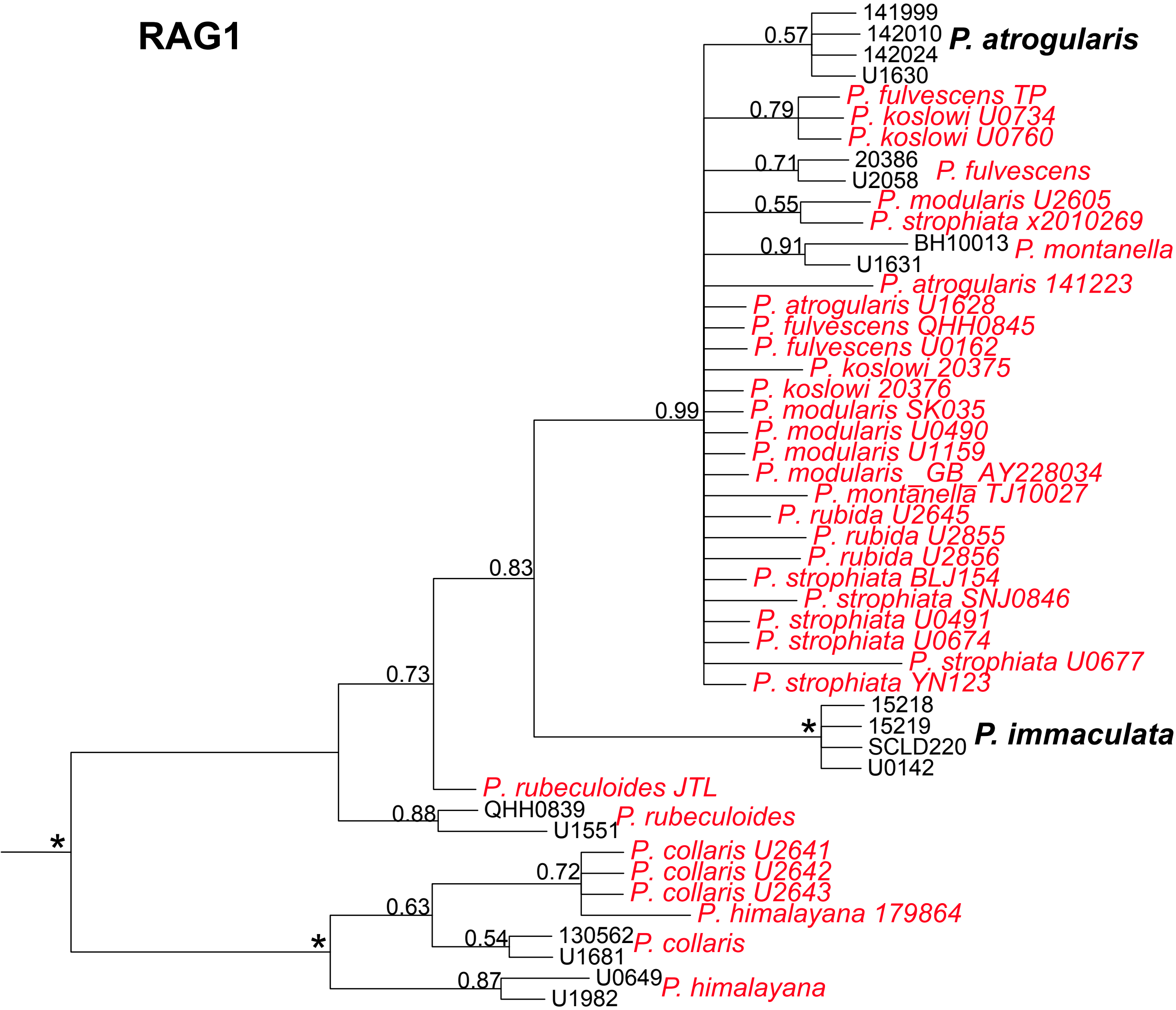

TGFB

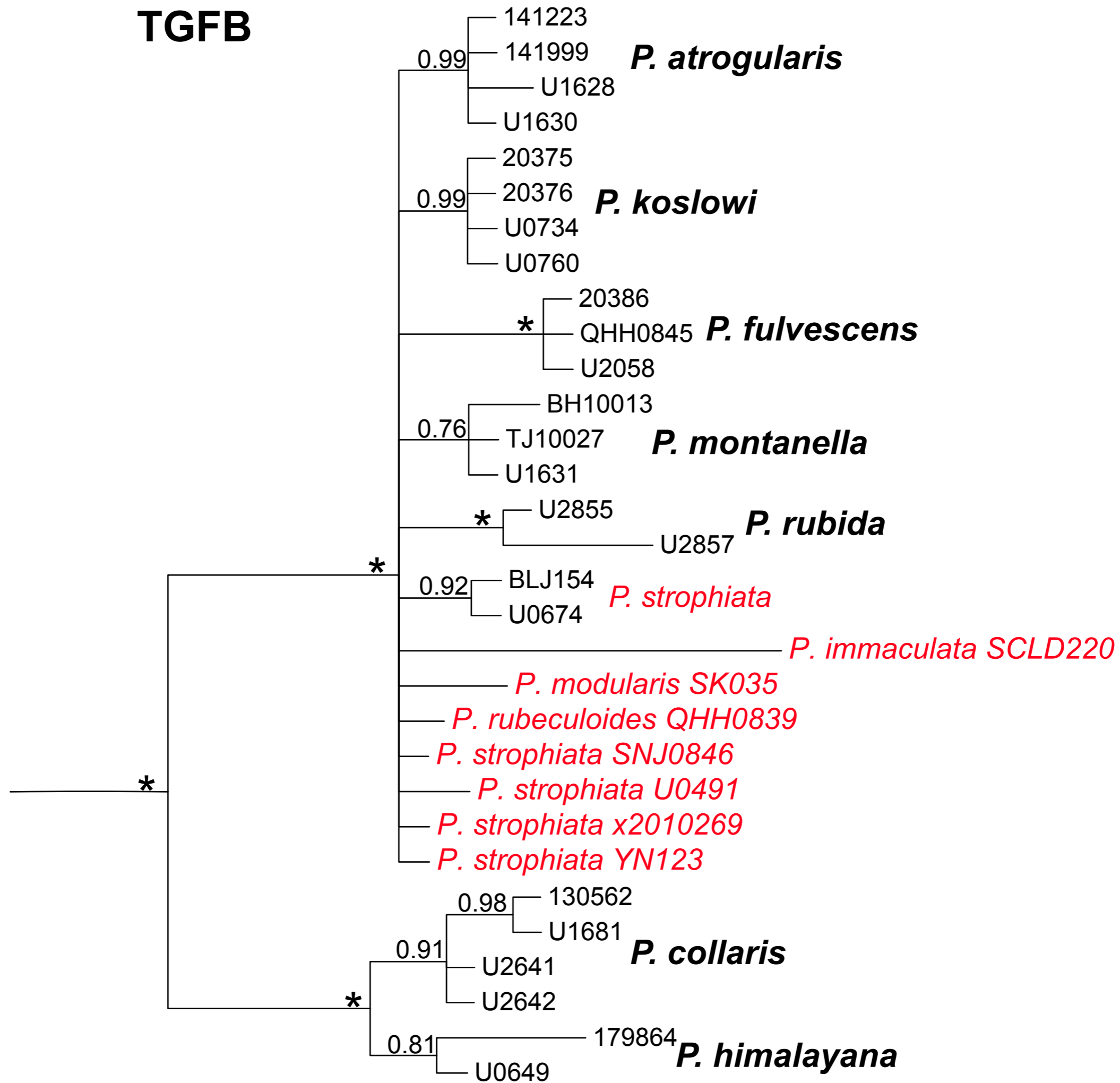

BRM

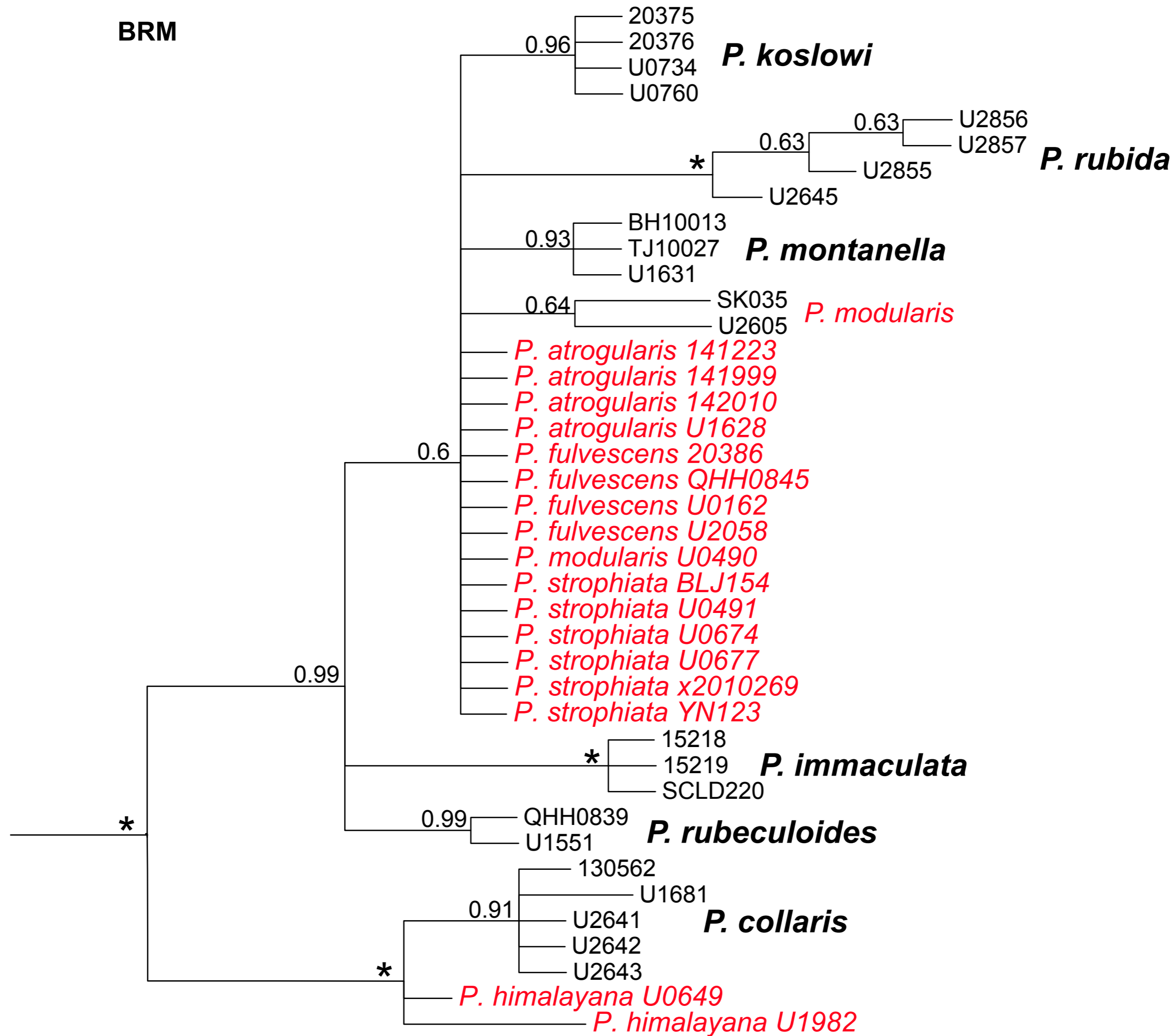

CHD1Z

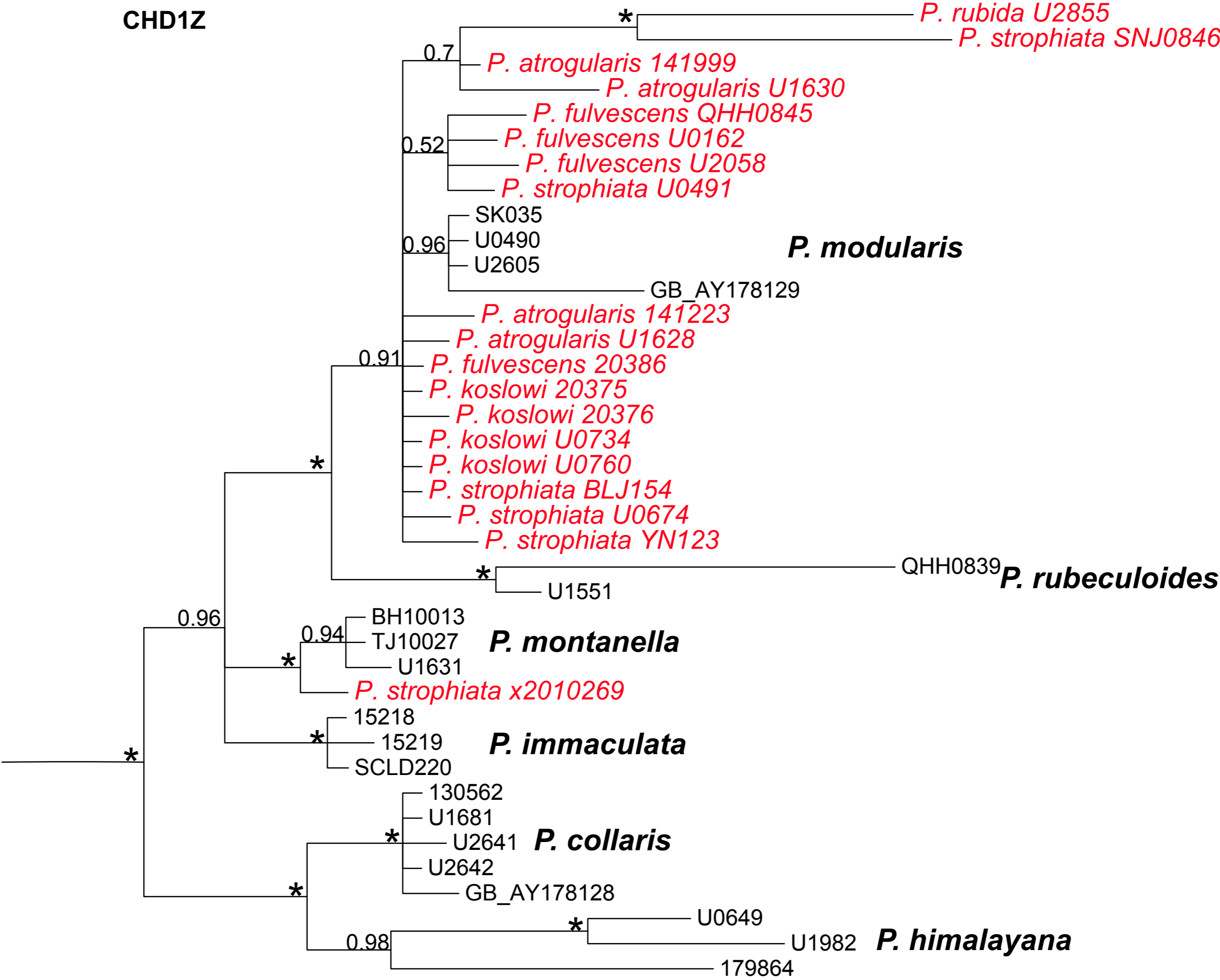

# Z6

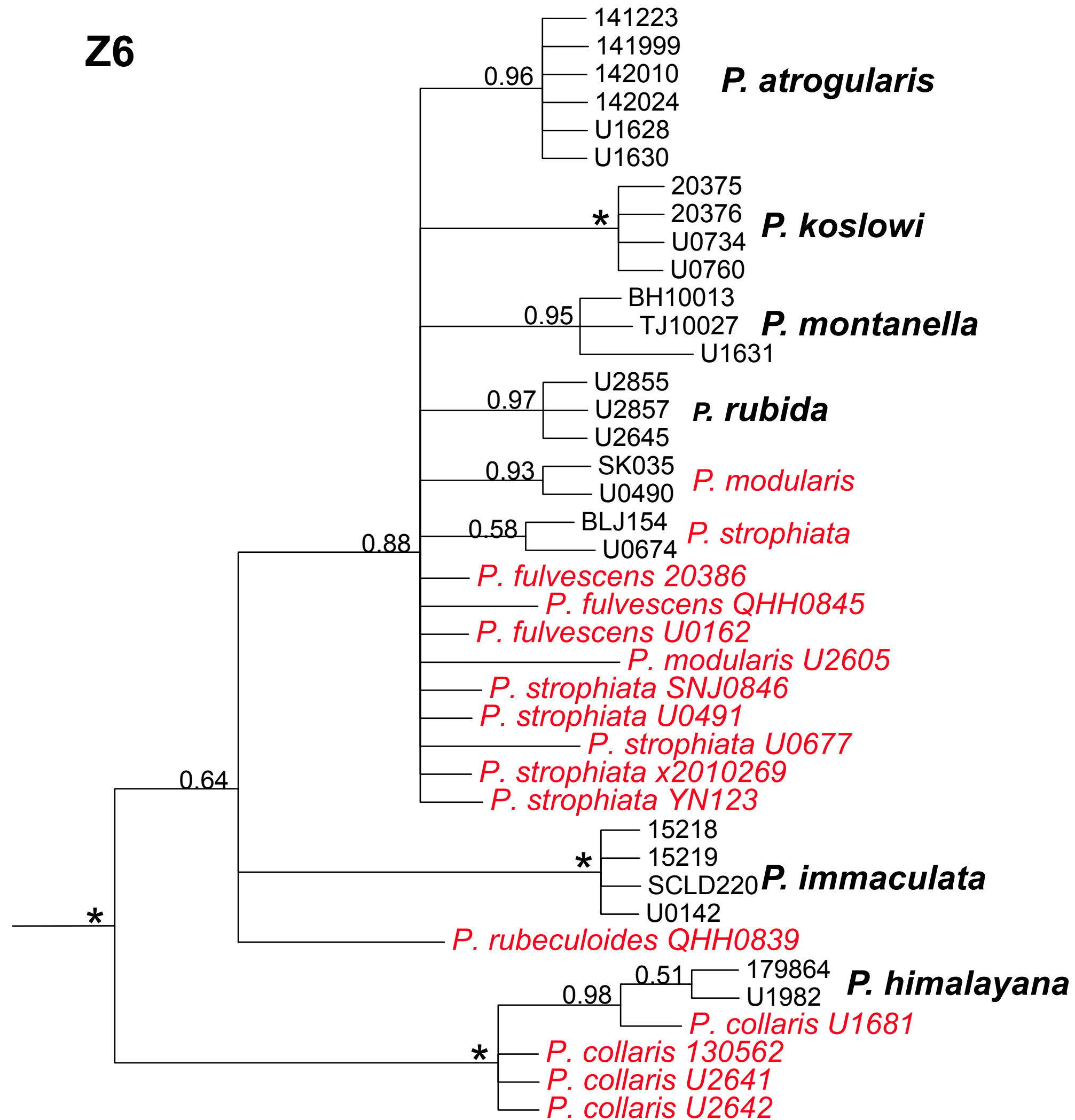

---

0.2

Locus 19

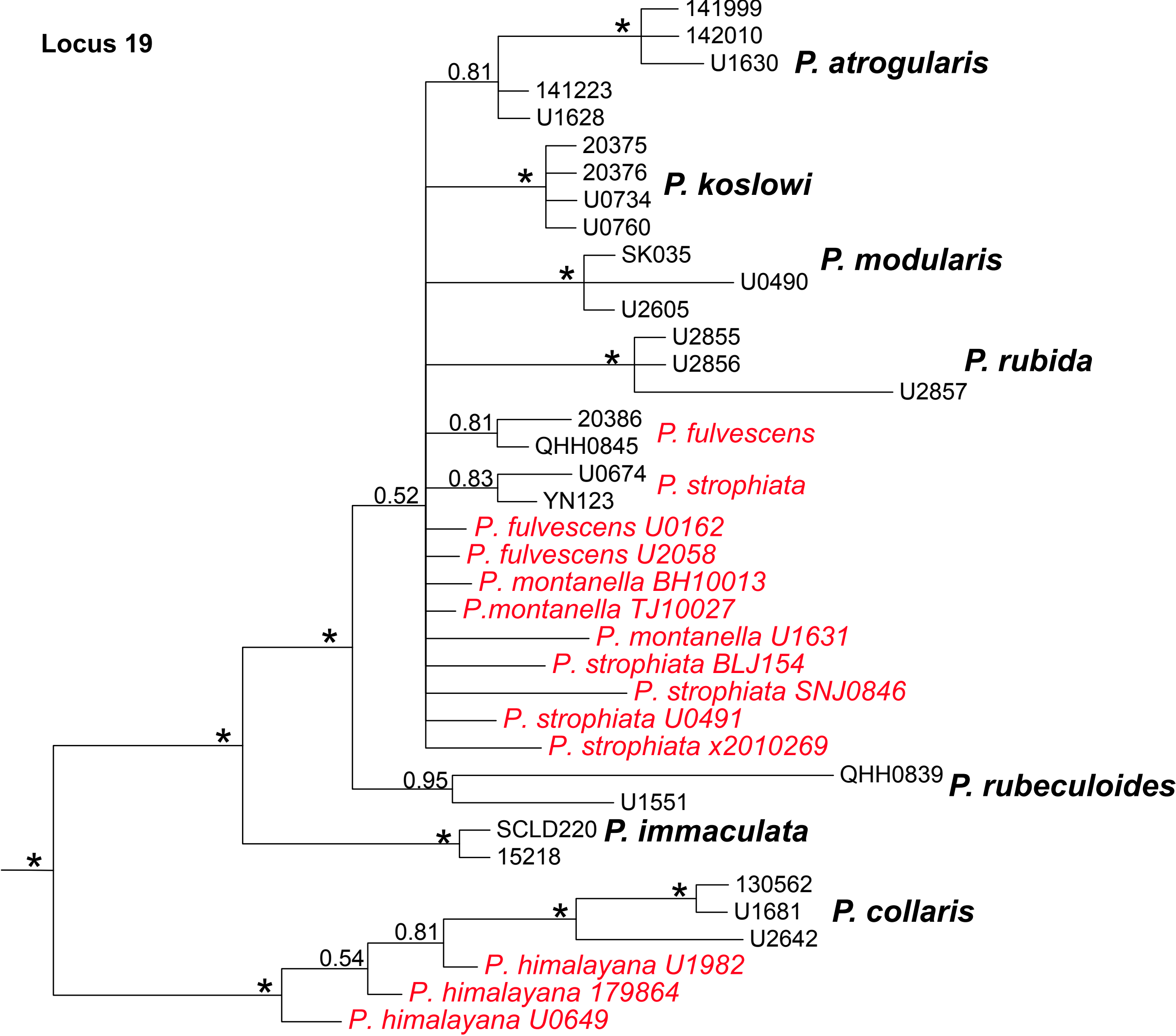

Supplement: Supplementary file 2 [file ECE3-7-6346-s002.pdf]

a  
Cytb + CO1

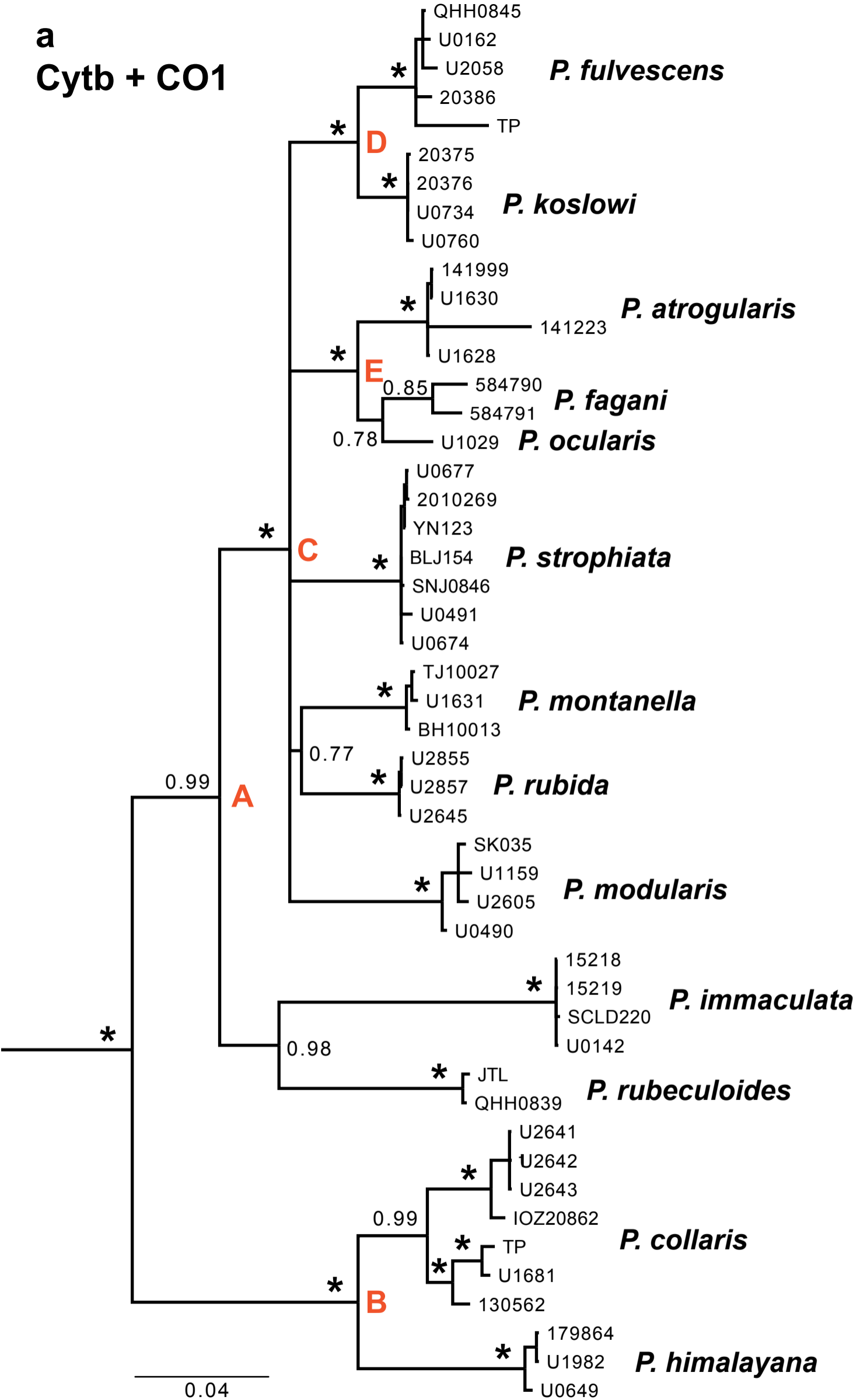

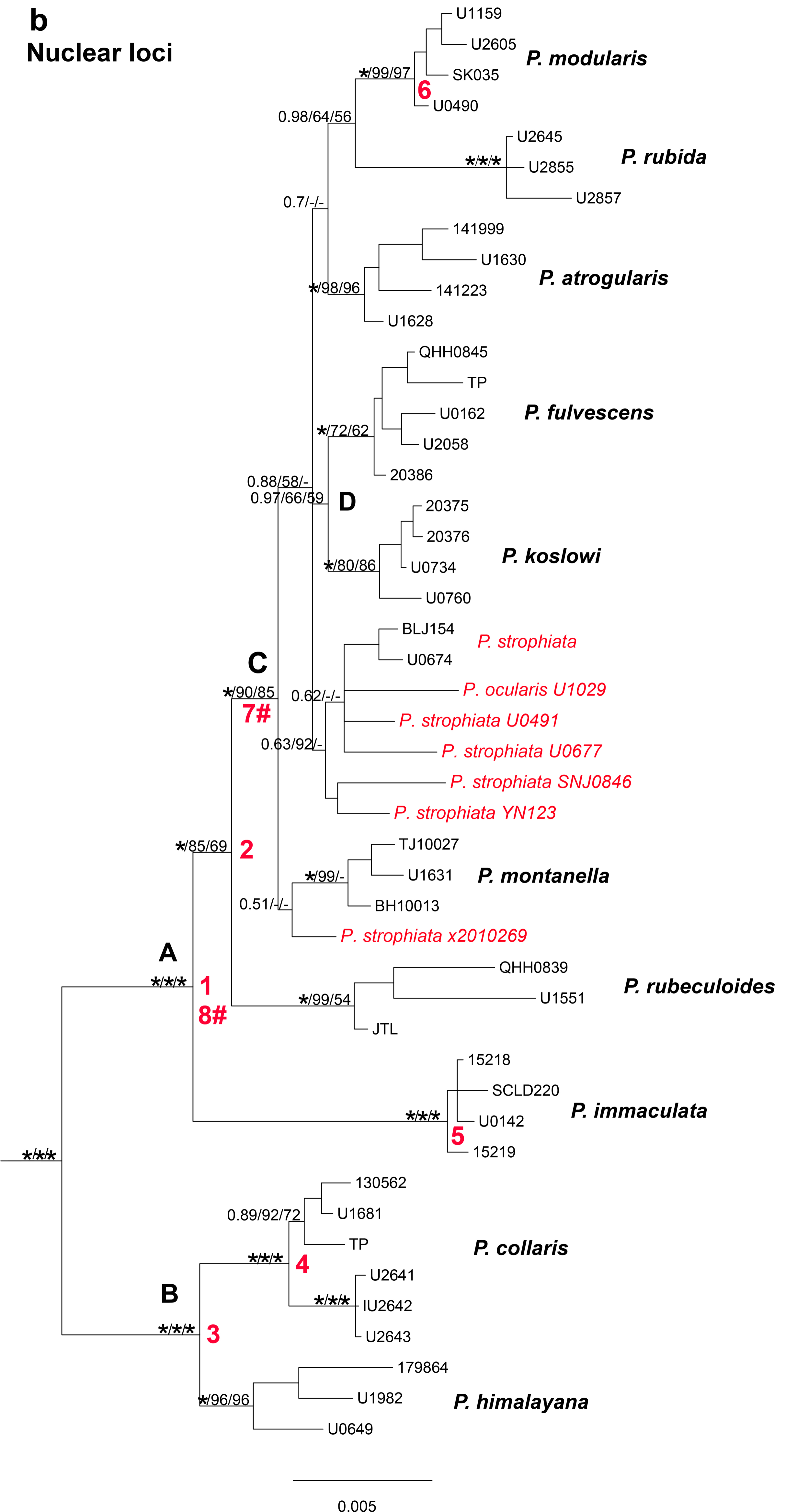

Supplement: Supplementary file 3 [file ECE3-7-6346-s003.pdf]

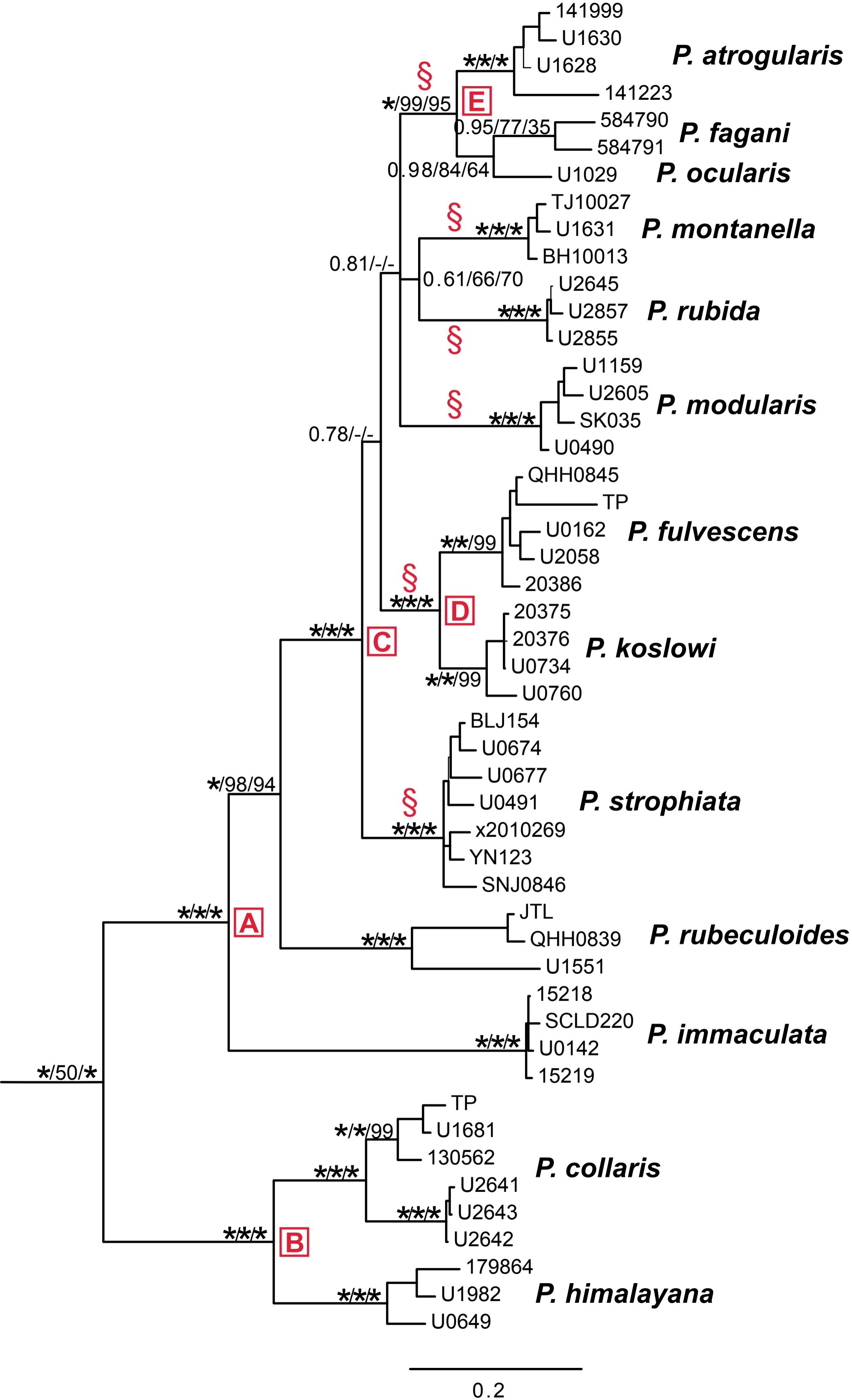

Supplement: Supplementary file 4 [file ECE3-7-6346-s004.pdf]

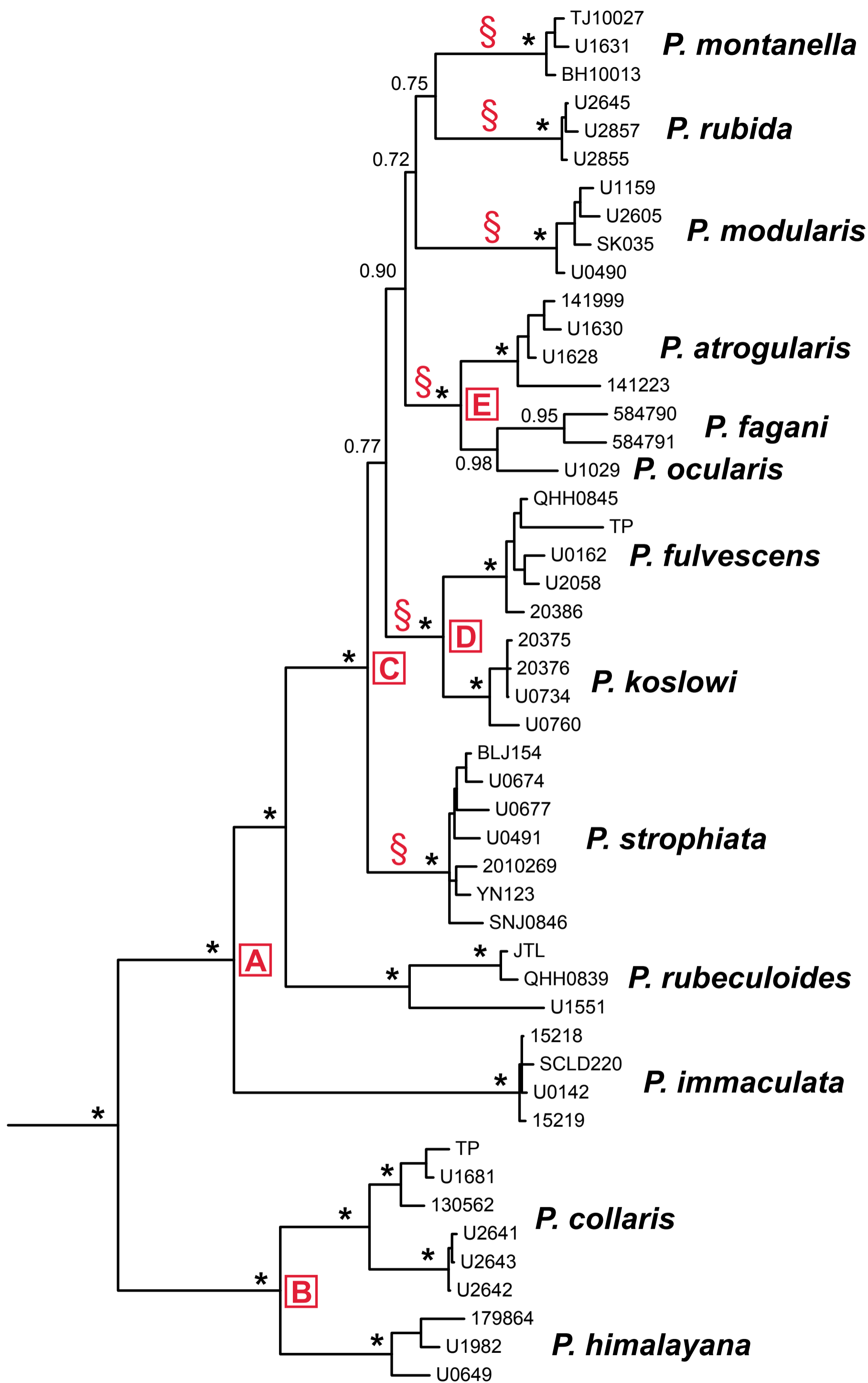

0.02

Supplement: Supplementary file 5 [file ECE3-7-6346-s005.pdf]

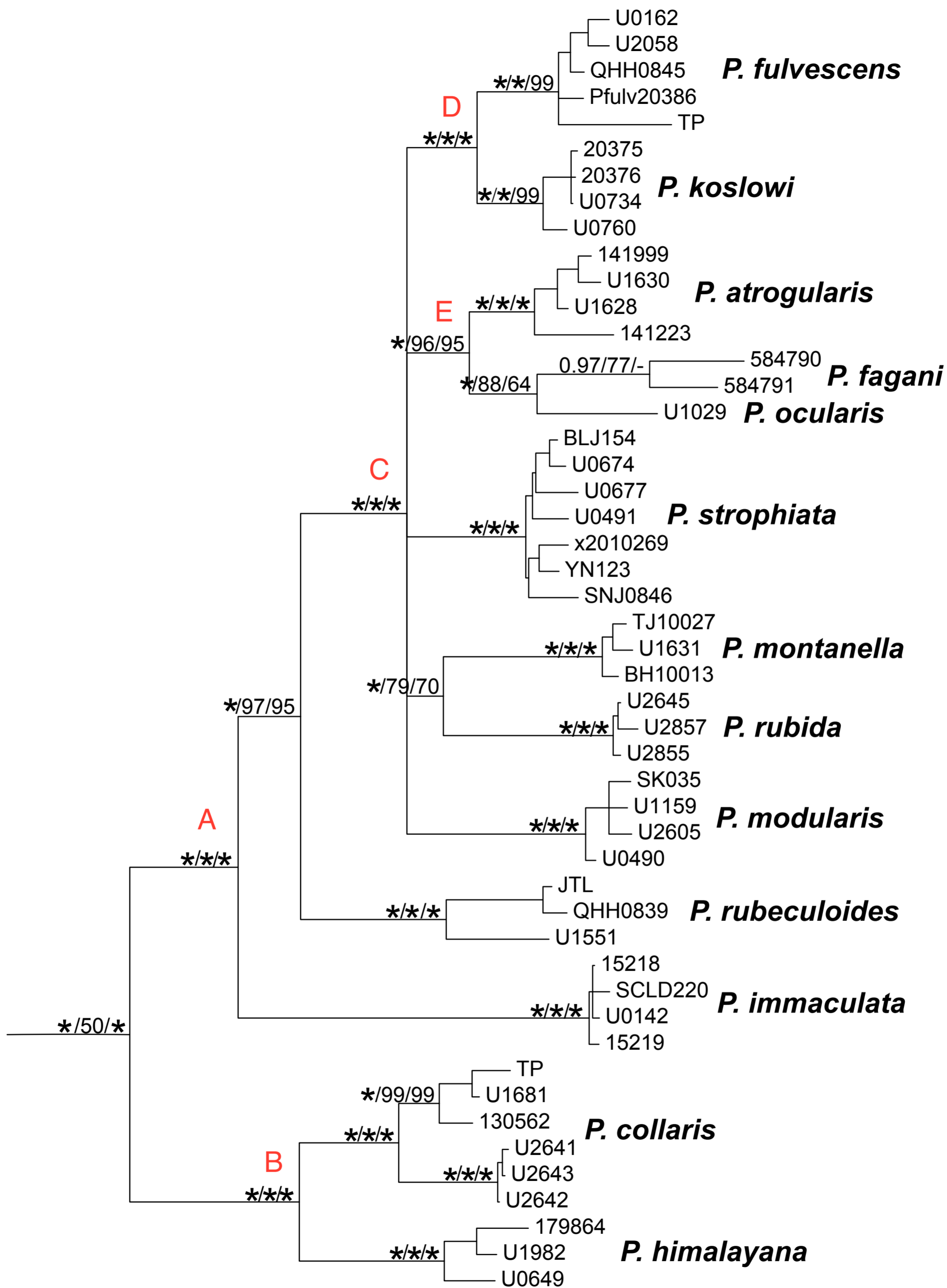

Supplement: Supplementary file 6 [file ECE3-7-6346-s006.pdf]

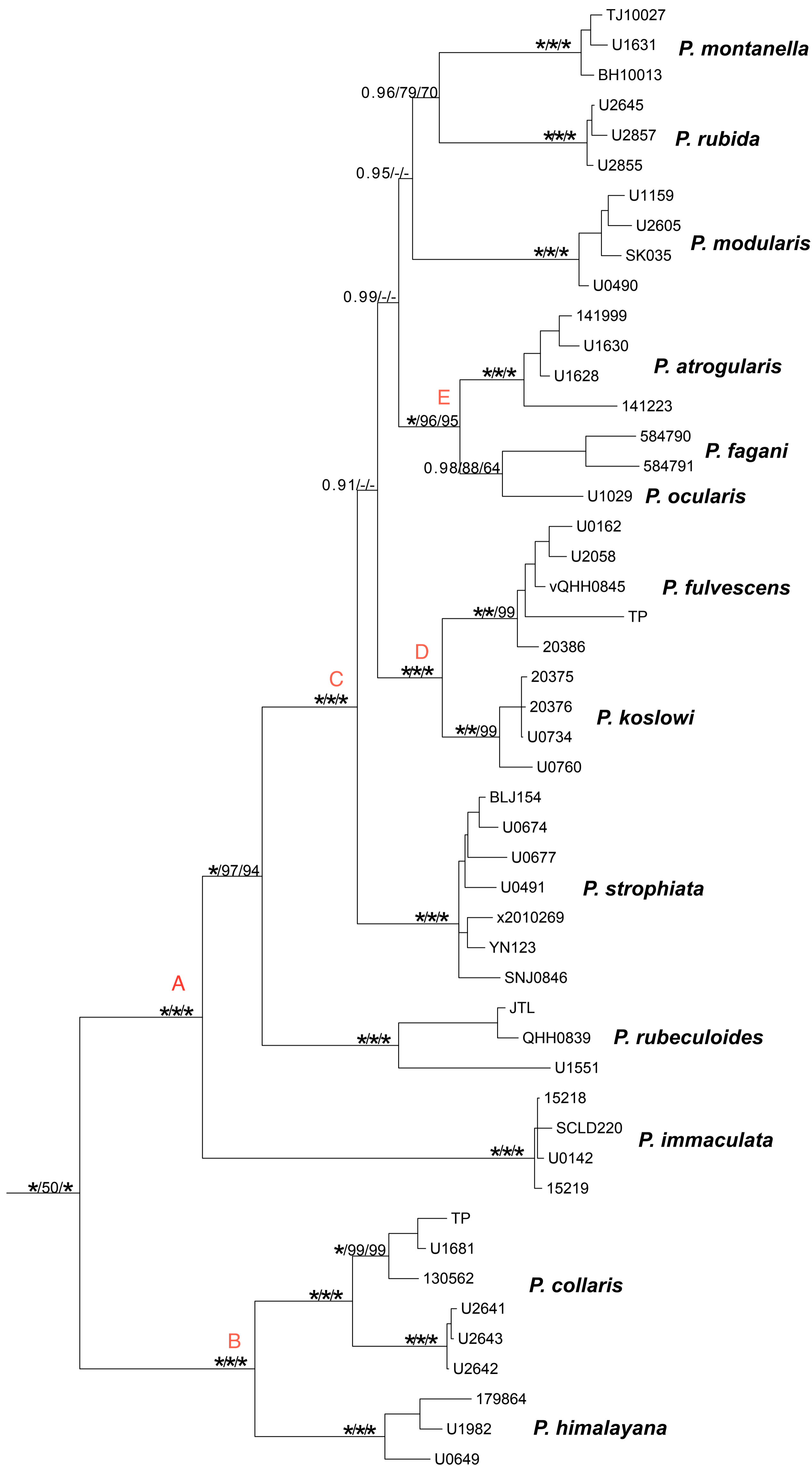

Supplement: Supplementary file 7 [file ECE3-7-6346-s007.pdf]

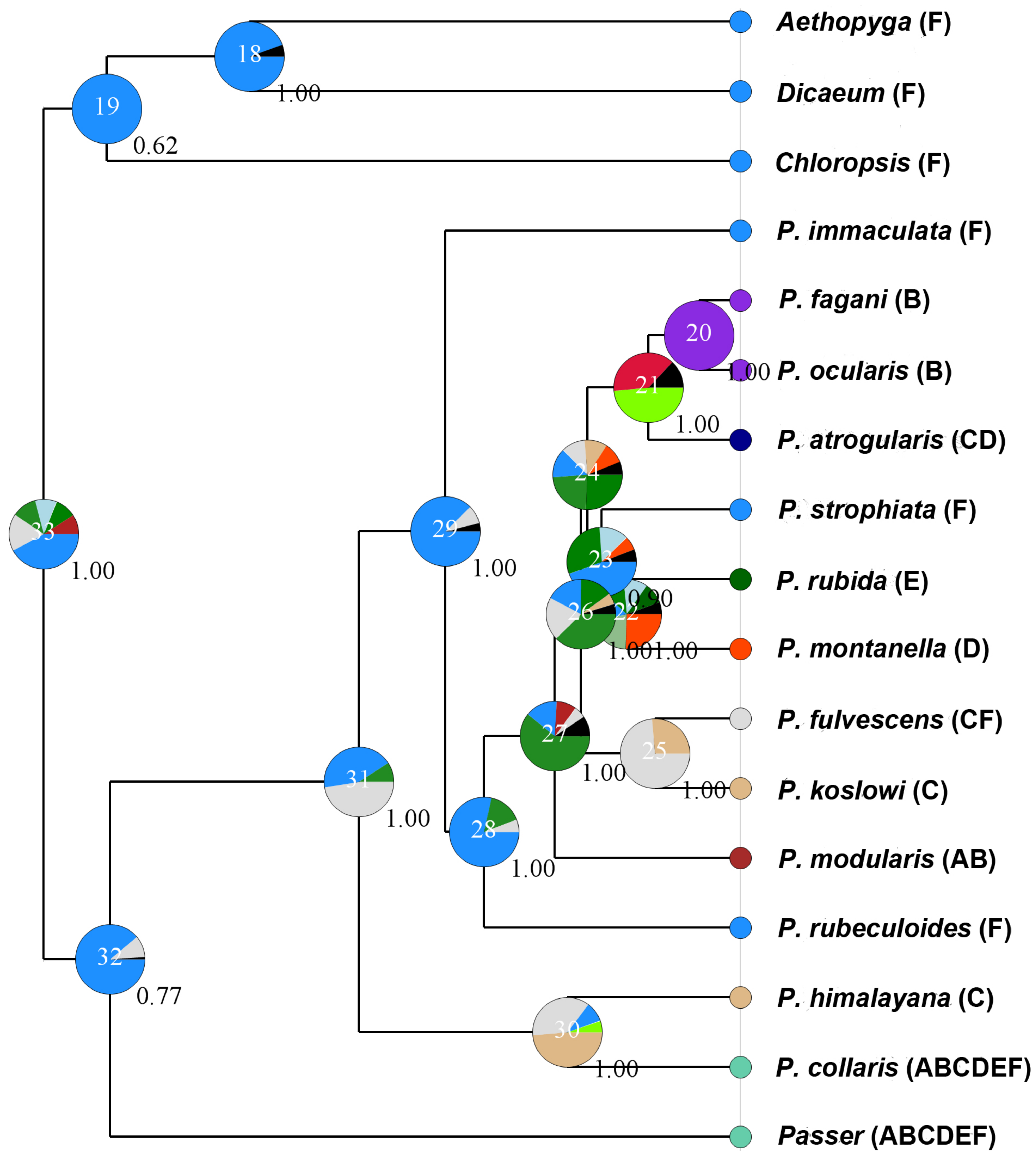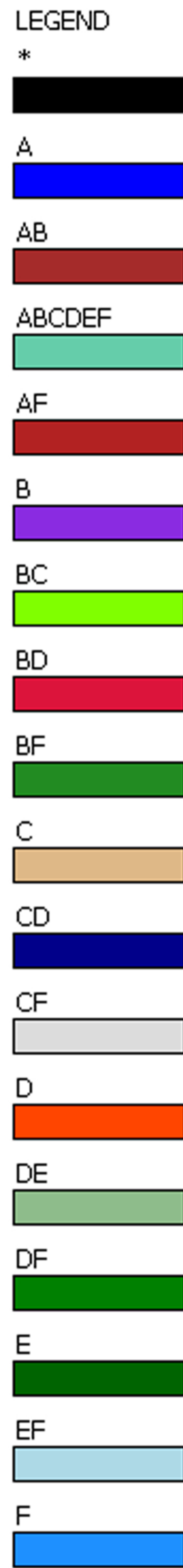

Supplement: Supplementary file 8 [file ECE3-7-6346-s008.pdf]

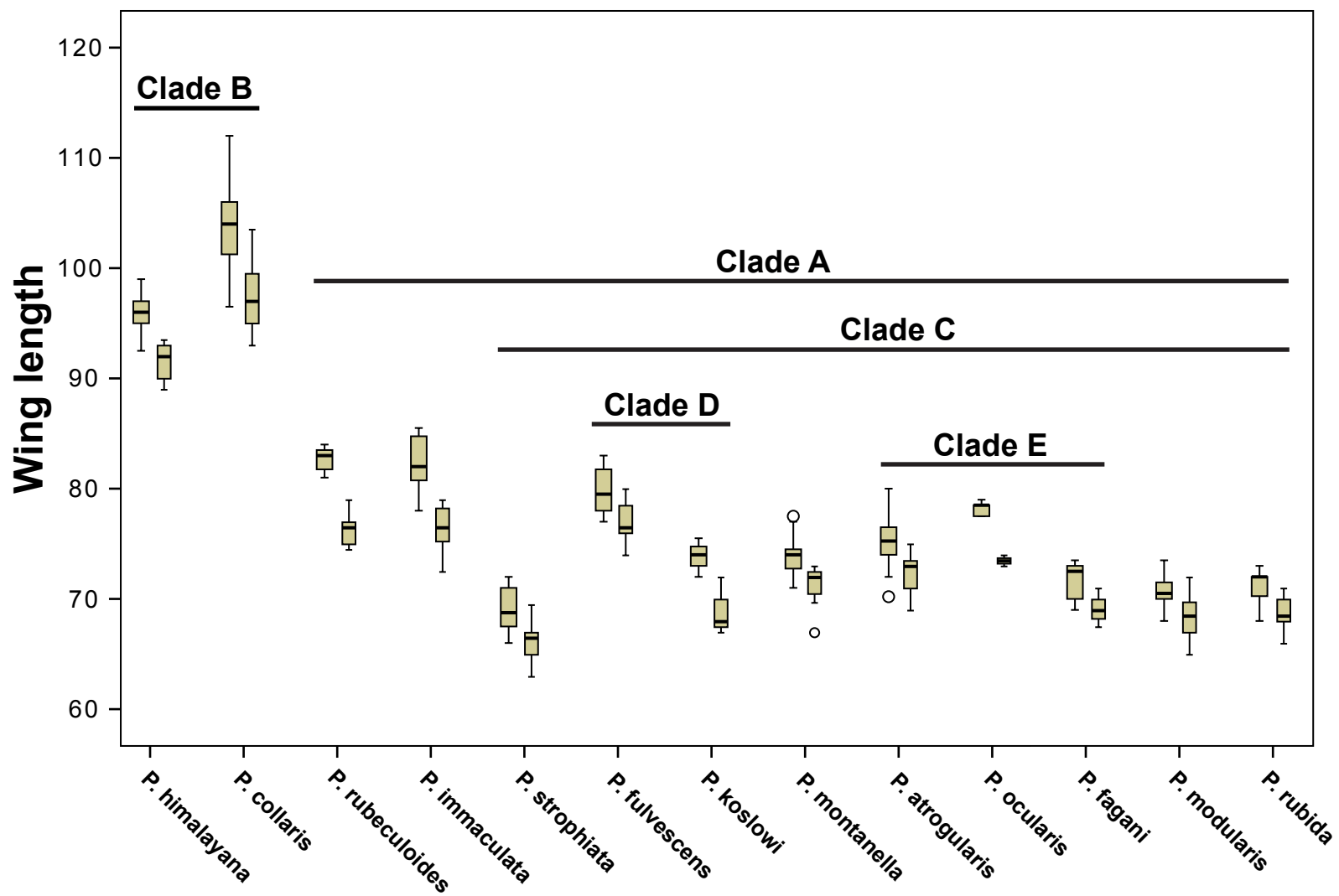

Supplement: Supplementary file 9 [file ECE3-7-6346-s009.pdf]

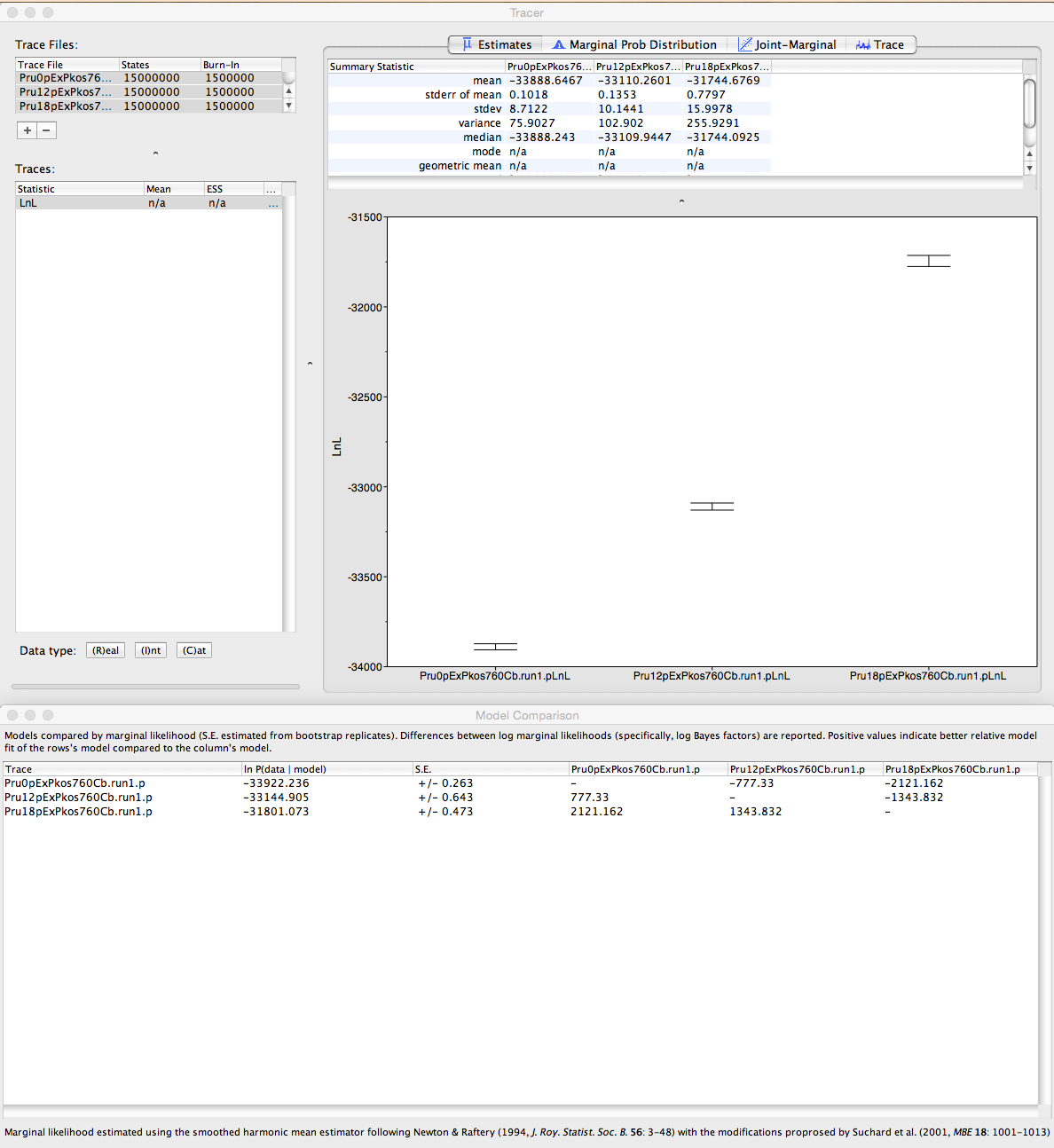

Supplement: Supplementary file 14 [file ECE3-7-6346-s014.tiff]
