# Supplementary material for: Explosive radiation and spatial expansion across the cold environments of the Old World in an avian family
Source: Ecol Evol. 2017 Jul 6;7(16):6346–57. doi: 10.1002/ece3.3136 (PMC5574758; doi:10.1002/ece3.3136)
Supplement: Supplementary file 12 [file ECE3-7-6346-s012.pdf]

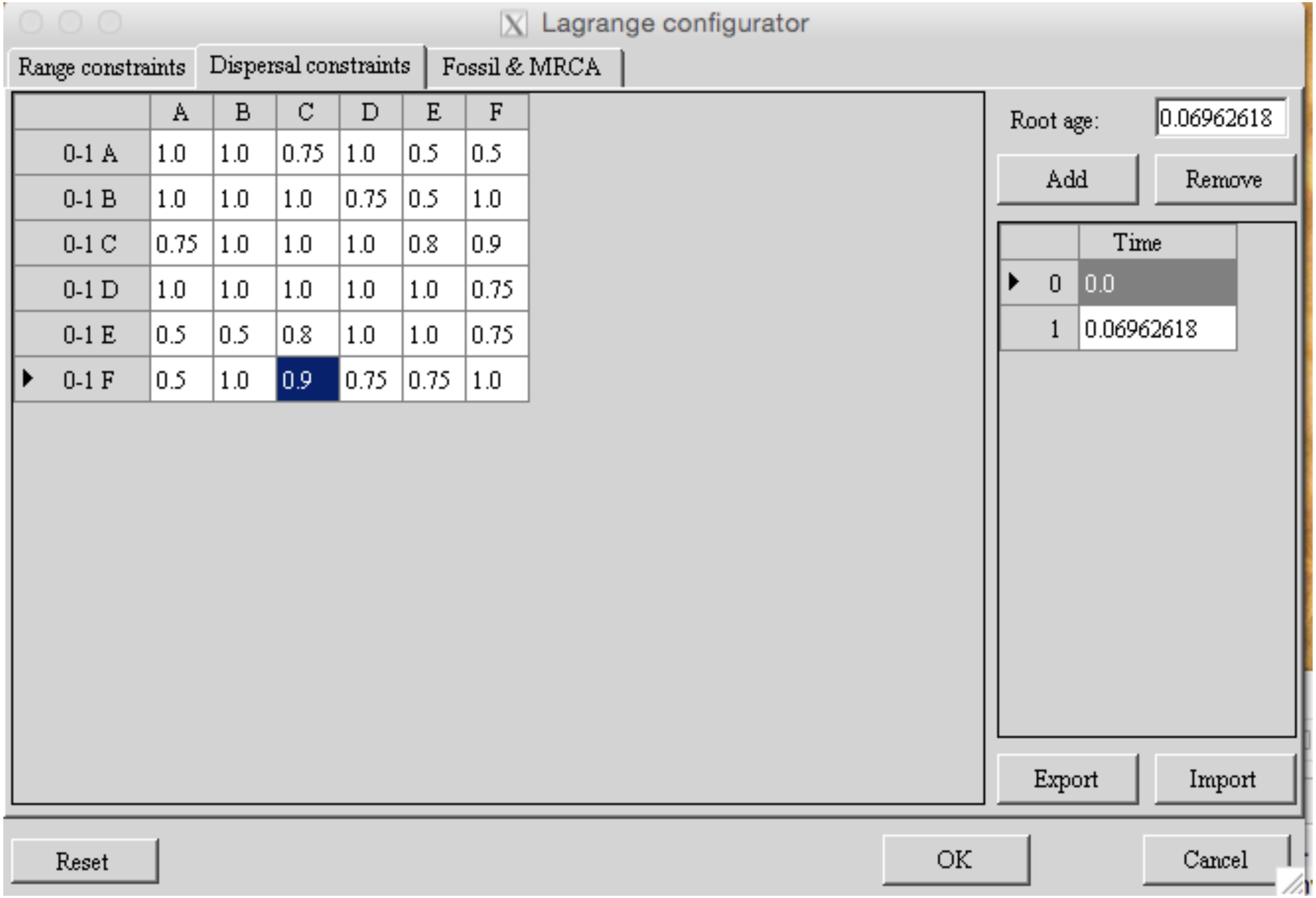
Lagrange configurator
Range constraints
Dispersal constraints
Fossil & MRCA
A B C D E F
0-1 A 1.0 1.0 0.75 1.0 0.5 0.5
0-1 B 1.0 1.0 1.0 0.75 0.5 1.0
0-1 C 0.75 1.0 1.0 1.0 0.8 0.9
0-1 D 1.0 1.0 1.0 1.0 1.0 0.75
0-1 E 0.5 0.5 0.8 1.0 1.0 0.75
0-1 F 0.5 1.0 0.9 0.75 0.75 1.0
Root age:
0.06962618
Add
Remove
Time
0 0.0
1 0.06962618
Export
Import
Reset
OK
Cancel
