# Supplementary material for: Explosive radiation and spatial expansion across the cold environments of the Old World in an avian family
Source: Ecol Evol. 2017 Jul 6;7(16):6346–57. doi: 10.1002/ece3.3136 (PMC5574758; doi:10.1002/ece3.3136)
Supplement: Supplementary file 15 [file ECE3-7-6346-s015.docx]

|  | *collaris* | *himalayana* | *rubeculoides* | *strophiata* | *montanella* | *fulvescens* | *fagani* | *ocularis* | *koslowi* | *atrogularis* | *rubida* | *immaculata* | *modularis* |
| --- | --- | --- | --- | --- | --- | --- | --- | --- | --- | --- | --- | --- | --- |
| *collaris* | – |  |  |  |  |  |  |  |  |  |  |  |  |
| *himalayana* | *** | – |  |  |  |  |  |  |  |  |  |  |  |
| *rubeculoides* | *** | n.s. | – |  |  |  |  |  |  |  |  |  |  |
| *strophiata* | *** | *** | *** | – |  |  |  |  |  |  |  |  |  |
| *montanella* | *** | *** | *** | ** | – |  |  |  |  |  |  |  |  |
| *fulvescens* | *** | n.s. | n.s. | *** | *** | – |  |  |  |  |  |  |  |
| *fagani* | *** | ** | n.s. | *** | n.s. | n.s. | – |  |  |  |  |  |  |
| *ocularis* | *** | ** | * | *** | n.s. | n.s. | n.s. | – |  |  |  |  |  |
| *koslowi* | *** | *** | * | n.s. | n.s. | *** | n.s. | n.s. | – |  |  |  |  |
| *atrogularis* | *** | *** | *** | *** | n.s. | *** | n.s. | n.s. | n.s. | – |  |  |  |
| *rubida* | *** | *** | *** | n.s. | n.s. | *** | n.s. | n.s. | n.s. | n.s. | – |  |  |
| *immaculata* | *** | *** | *** | *** | n.s. | *** | n.s. | n.s. | n.s. | n.s. | n.s. | – |  |
| *modularis* | *** | *** | *** | n.s. | n.s. | *** | * | n.s. | n.s. | * | n.s. | * | – |

**Supplementary Table S6.** One-way ANOVA followed by Tukey’s post hoc test of PC1 from PCA based on morphometrics (cf. Figure 5).
